# Theory: Treatments for Prolonged ICU Patients May Provide New Therapeutic Avenues for Myalgic Encephalomyelitis/Chronic Fatigue Syndrome (ME/CFS)

**DOI:** 10.3389/fmed.2021.672370

**Published:** 2021-05-07

**Authors:** Dominic Stanculescu, Lars Larsson, Jonas Bergquist

**Affiliations:** ^1^Independent Researcher, Sint Martens Latem, Belgium; ^2^Basic and Clinical Muscle Biology, Department of Physiology and Pharmacology, Karolinska Institute, Solna, Sweden; ^3^Analytical Chemistry and Neurochemistry, Department of Chemistry–Biomedical Center, Uppsala University, Uppsala, Sweden; ^4^The Myalgic Encephalomyelitis/Chronic Fatigue Syndrome (ME/CFS) Collaborative Research Centre at Uppsala University, Uppsala, Sweden

**Keywords:** ME/CFS, treatment, suppressed endocrine axis, prolonged critical illness, oxidative and nitrosative stress, chronic critical care illness, non-thyroidal illness syndrome, post viral fatigue syndrome

## Abstract

We here provide an overview of treatment trials for *prolonged* intensive care unit (ICU) patients and theorize about their relevance for potential treatment of myalgic encephalomyelitis/chronic fatigue syndrome (ME/CFS). Specifically, these treatment trials generally target: (a) the correction of suppressed endocrine axes, notably through a “reactivation” of the pituitary gland's *pulsatile* secretion of tropic hormones, or (b) the interruption of the “vicious circle” between inflammation, oxidative and nitrosative stress (O&NS), and low thyroid hormone *function*. There are significant parallels in the treatment trials for *prolonged* critical illness and ME/CFS; this is consistent with the hypothesis of an overlap in the mechanisms that prevent recovery in both conditions. Early successes in the simultaneous reactivation of *pulsatile* pituitary secretions in ICU patients—and the resulting positive metabolic effects—could indicate an avenue for treating ME/CFS. The therapeutic effects of thyroid hormones—including in mitigating O&NS and inflammation and in stimulating the adreno-cortical axis—also merit further studies. Collaborative research projects should further investigate the lessons from treatment trials for *prolonged* critical illness for solving ME/CFS.

## Introduction

Critical illness refers to the physiological response to virtually any *severe* injury or infection, such as sepsis, liver disease, HIV infection, SARS-CoV-2 infection, head injury, pancreatitis, burns, cardiac surgery, etc. generally resulting in intensive care unit (ICU) hospitalization (1). *Prolonged* or chronic critical illness—a term applied to patients that survive *severe* injury or infection but fail to start recovering after a few days—is characterized by the suppression of multiple endocrine axes, irrespective of the nature of the original infection or trauma (2–7). This endocrine suppression is, however, not readily observable in single or average measurements of circulating tropic and non-tropic hormone concentrations (which are a function of both hormone release and elimination from the blood stream); instead measurements of the *frequency* and *amplitude* of pituitary secretions (i.e., of the *pulsatility*) performed on ICU patients as often as every 10 min over 24 h are required to reveal the endocrine suppression (8). Pro-inflammatory cytokines play a role in inducing and maintaining the uniform suppression of the endocrine axes during *prolonged* critical illness—predominantly at the level of the hypothalamus and pituitary (9–15). Moreover, reciprocal relationships between cytokines, oxidative and nitrosative stress (O&NS), and thyroid hormone *function* appear to perpetuate illness (c.f. “vicious circle”) (13, 16). These patterns are increasingly recognized as maladaptive and inhibiting patients' recovery, thus requiring treatment independent of the initial infection or trauma (5, 17–19). Moreover, it has been suggested that the persistence of the endocrine disturbances could also explain “post-intensive care syndrome (PICS)” (20); i.e., “the cognitive, psychiatric and /or physical disability after treatment in ICUs,” including ICU-acquired weakness (21).

ME/CFS is a debilitating, multi-system disease of unclear etiology (22, 23). The most common peri-onset events reported by patients are infection-related episodes, stressful incidents, and exposure to environmental toxins (24). “Impaired function, post-exertional malaise (an exacerbation of some or all of an individual's ME/CFS symptoms after physical or cognitive exertion, or orthostatic stress that leads to a reduction in functional ability), and unrefreshing sleep” are considered to be core symptoms (25, 26). The severity of the symptoms varies: “very severely affected patients experience profound weakness, almost constant pain, severe limitations to physical and mental activity, sensory hypersensitivity (light, touch, sound, smell, and certain foods), and hypersensitivity to medications” (27). The disease can be completely incapacitating: “at least one-quarter of ME/CFS patients are house- or bedbound at some point in their lives” (25). Patients with milder symptoms experience a significant reduction in previous levels of functioning (23). The disease progresses over time: similar to critical illness, an early hypermetabolic state may culminate in a hypometabolic state with low energy production (28). The progressive nature of the disease makes establishing a set of diagnostic criteria or molecular markers particularly difficult. There are currently no effective treatments for ME/CFS (29–33).

In a previous publication (34) we argued that—without excluding possible predisposing genetic or environmental factors—the maladaptive mechanisms that prevent recovery in some ICU patients may also underlie ME/CFS. Specifically, these mechanisms are: (a) suppression of the pituitary gland's *pulsatile* secretion of tropic hormones, and (b) a “vicious circle” between inflammation, O&NS, and low thyroid hormone *function*. Here we provide an overview of past treatment trials for *prolonged* ICU patients which specifically address these mechanisms. We relate similar experimental trials to improve outcomes in ME/CFS in order to highlight the similarities in the treatment approaches for both conditions. As part of this overview we also draw on findings from fibromyalgia because ME/CFS and fibromyalgia are often jointly considered in the literature (35, 36); fibromyalgia is similarly a syndrome that is medically unexplained, often comorbid with ME/CFS, and “shares the core symptoms of fatigue, sleep problems, and cognitive difficulties” (37). Finally, we also suggest potential lessons to be learned from treatment trials for *prolonged* critical illness for the quest to solve ME/CFS.

The lessons-learned from the field of critical care medicine for ME/CFS may also be particularly relevant for the aftermath of the COVID-19 pandemic. Coronaviruses are associated with persistent inflammation (38) and endocrine dysfunctions (39)—elements that are central to both the pathologies of *prolonged* critical illness and ME/CFS. Many COVID-19 patients continue to experience a variety of debilitating symptoms despite successfully defeating the virus—termed “post COVID-19 syndrome” or “long COVID-19”– that resembles ME/CFS (40–45). Moreover, a recent analysis has shown that ME/CFS patients and COVID-19 recovery patients “share molecular signatures”—evidence of overlaps in immune and metabolic dysregulation (46).

## Compensation for and Correction of Suppressed Endocrine Axes

Researchers have tried to correct suppressed endocrine axes in *prolonged* critically ill patients with the hope of reducing muscle wasting and mortality, and to aid recovery. The treatment trials can be grouped into two main approaches: (A) treatments with non-tropic *peripheral* hormones, and (B) the “reactivation” of the *central* endocrine glands. Whereas, the first approach compensates for suppressed endocrine axes by providing down-stream hormones into circulation, the second approach attempts to correct the endocrine axes themselves through interventions at the central level. We briefly summarize various treatment trials for each of these two broad approaches below. For each approach we also relate similar experimental treatment trials for ME/CFS and fibromyalgia in order to highlight the similarities in the quests to cure the two conditions, and to derive lessons for solving ME/CFS.

### Approach A: Treatments With Peripheral Hormones

The use of *peripheral* hormones—notably glucocorticoids and other adrenal hormones, growth hormone (GH), insulin-like growth hormone-1 (IGF-1), thyroid hormones, and a combination of these hormones—have been trialed to improve outcomes in *prolonged* critical illness as well as in ME/CFS and fibromyalgia with varied successes as described below.

#### Treatments With Glucocorticoids and Other Adrenal Hormones

##### In Prolonged Critical Illness

Administration of large daily doses of hydrocortisone (200–300 mg) in patients during critical illness is quite common, particularly in sepsis or when cortisol levels are deemed low relative to the severity of the illness. The aim is to treat “critical illness-related corticosteroid insufficiency” (CIRCI) which is thought to “lead to an exaggerated proinflammatory response with increased tissue injury and organ dysfunction” (9, 47). Some researchers, however, have recently argued that such high doses of hydrocortisone may be counterproductive because they drive the inhibitory feedback loop inherent to endocrine axes, resulting in further central suppression of the axes (19). Moreover, large hydrocortisone doses heighten *catabolic* effects (i.e., the break-down of molecules and tissues), especially if administered for too long [see review in (5, 48)].

##### In ME/CFS

Low production of adrenal hormones (i.e., partial hypoadrenalism) has been well-documented in ME/CFS (49–66). Several studies showed that a low dose of hydrocortisone could benefit ME/CFS patients, notably in reducing fatigue and disability scores [see reviews in (67, 68)]. Daily doses of 5–15 mg of hydrocortisone appear not to further suppress the adreno-cortical axis (50, 69)—also called hypothalamic-pituitary-adrenal (HPA) axis—and may even improve the otherwise “blunted” responses of the pituitary to the signal from the hypothalamus (56). However, researchers have revealed that somewhat higher doses of hydrocortisone (25–35 mg per day) lead to a moderate decrease in endogenous adrenocorticotropic hormone (ACTH) and cortisol production in ME/CFS patients, via the negative feedback loop (50, 70). Consequently, there has been a debate since the late 1990s between researchers who argue that—despite improvement in symptoms—“adrenal suppression precludes the practical use of hydrocortisone” for ME/CFS patients (70), and those who stress that at low physiological doses hydrocortisone treatment for ME/CFS is safe and effective (67, 71–78).

The effects of supplementation with *other* adrenal hormones on ME/CFS symptoms has also been studied. Fludrocortisone (a corticosteroid) led to positive results in some trials (79–81), but not in others (82–84). In an uncontrolled pilot-study the supplementation with DHEA (an adrenal hormone with anabolic properties) led to a significant reduction in ME/CFS symptoms, including pain, fatigue, helplessness, anxiety, memory loss, and sexual problems (85). Finally, a recent study suggested pregnenolone sulfate (an endogenous neurosteroid derived from the adrenal hormone pregnenolone) may have therapeutic potential to restore the Transient Receptor Potential Melastatin 3 (TRPM3) ion channel function in natural killer cells in ME/CFS (86).

In summary, glucocorticoids and other adrenal hormones are used in practice to compensate for lower endogenous hormone production in both *prolonged* critical illness and ME/CFS. Treatments aim to manage inflammation and improve symptoms. However, their use is questioned by researchers in both fields because they tend to reinforce central HPA axis suppression.

#### Treatments With GH and IGF-1

##### In Prolonged Critical Illness

The hormone IGF-1 has been tested and applied in critical illness for decades, with positive results in reducing catabolism (i.e., muscle and protein loss), recovery of gut mucosal function, tissue repair, control over inflammatory cytokines, decreased protein oxidation, increased glucose oxidation, etc. [see review (87)]. However, research has shown that to avoid side effects doses must be physiological (i.e., not higher than regularly produced by the body). Some positive results have also occurred with administration of GH (or the synthetic version, rhGH) during critical illness [see reviews (87, 88)]. However, a large-scale double-blind randomized control study of rhGH infusions undertaken in 1999 resulted in increased mortality of critically ill patients (89). This led to the near cessation of the use of GH or rhGH in critical care. Other researchers argue that dosages in this study were too high, thereby overwhelming the negative feedback loops inherent to endocrine axes maintaining homeostasis (88). Finally, some promising trials have also been performed combining GH and IGF-1 in critical illness (90, 91). GH and IGF-1 have complementary roles in the balance between anabolic (i.e., the building of molecules and tissue) and catabolic activities (91).

##### In ME/CFS

There is also evidence for low GH secretion in ME/CFS (92, 93). A small placebo-controlled study found that treatment with GH injections in ME/CFS patients over 12 months was beneficial: a few of the patients were even able to resume work after long periods of sick leave (94). Evidence of relative GH deficiency in fibromyalgia (95–101) also spurred a series of placebo-controlled studies which demonstrated that GH injections over several months—in the form of physiological doses or doses adapted to increase IGF-1 to a specific level—reduced pain and improved quality of life scores in fibromyalgia patients (94, 102–105).

In summary, GH and IGF-1 have been trialed for both *prolonged* critical illness and ME/CFS with reports of beneficial outcomes. However, their use is not common in practice, notably because of the risks involved. Supplementation with these hormones also does not serve to relieve central endocrine axis suppression but would rather reinforce it.

#### Treatments With Thyroid Hormones

##### In Prolonged Critical Illness

Clinicians already began in the late 1970s to suggest thyroid hormone supplementation for their critical patients in an attempt to increase survival rates (106–108). This came out of a realization that these patients suffered from a depressed level of thyroid hormone activity independent of the health of the thyroid gland—termed “non-thyroidal illness syndrome (NTIS),” “euthyroid sick syndrome” or “low T3 syndrome” (109, 110). Supplementation of thyroid hormone during critical illness continues to be the subject of intense debate (111–115). Results with thyroid hormone supplementation have been mixed [see reviews (116–118)]. The type of supplement (synthetic T4 or T3), the timing of treatment and the dosage could explain the variable, but often poor outcomes. Specifically, given that thyroid hormone conversion from its “*inactive*” (T4) to “*active*” (T3) form by deiodinase enzymes is impaired during illness through the actions of pro-inflammatory cytokines (13, 16, 114, 119, 120), it has been suggested that T3 supplementation may be more effective than T4 supplementation (121–123). In fact, given that T4 *up-regulates* the enzyme (deiodinase D3) which converts thyroid hormones into “*inactivated*” forms and *down-regulates* the enzyme (D2) responsible for thyroid hormone conversion to the “*active*” forms (124, 125), any T4 supplementation could exasperate NTIS. Yet, because of the short half-life of T3 and its normal circadian rhythm (126), the timing and periodicity of any T3 administration is likely to be an important determinant of its effect (127, 128). Finally, recognizing the fact that thyroid hormone uptake (i.e., transport into cells and binding by cellular receptors) is downregulated in tissue-specific ways during critical illness (13, 113, 119, 120, 129), thyroid hormone supplementation doses may have to be supra-physiological to achieve results (130, 131). In conclusion, “at present, no evidence-based consensus or guideline advocates thyroid hormone treatment of NTIS in patients who are critically ill,” (117) but new calculated parameters derived from mathematical modeling of thyroid hormone *function* may in the future assist in identifying better therapeutic thyroid hormone interventions in patients (132).

##### In ME/CFS

Recent studies suggest the existence of low thyroid hormone *function* in ME/CFS—i.e., low impact of thyroid hormone on target glands or tissues despite “normal” TSH and T4 lab results (133, 134). Thus, thyroid hormone *function* in ME/CFS resembles the “euthyroid sick syndrome” (NTIS) described in the field of critical medicine (34).

In small placebo-controlled studies in the 1990s, Lowe et al., showed that supraphysiologic dosages of T3 (75–150 mcg/day) were safe and significantly effective in the treatment of fibromyalgia: “significant improvement in clinical symptoms were recorded in T3 phases compared to baseline and placebo phases” (135–138). Given that patients had been euthyroid (i.e., their serum TSH and T4 values did not indicate hypothyroidism), Lowe suggested that “euthyroid fibromyalgia is a clinical phenotype of partial peripheral resistance to thyroid hormone” (135, 139) (i.e., the uptake of thyroid hormones by transporters and receptors in cells is disturbed). In a subsequent placebo-controlled study, Teitelbaum et al. showed that euthyroid ME/CFS and fibromyalgia patients treated with T4 (Synthroid) or naturally desiccated thyroid hormone (Armor Thyroid)—in addition to adrenal hormones, vitamins, minerals, and antibiotics—also experienced significant improvements (140).

Moreover, for decades practitioners have been treating euthyroid patients suffering from ME/CFS and fibromyalgia symptoms with thyroid hormones (67, 73–78, 141–146); and several patients have written books to share their recovery stories (127, 147, 148). The treatments vary in the type of thyroid hormones used (e.g., natural desiccated thyroid, T3, or T4), the dosage (e.g., supra-physiological or physiological), the timing (e.g., circadian method, slow-release, or single dose) as well as the prescribed complementary vitamin and mineral supplements (149). Several practitioners emphasize the importance of providing adrenal hormones in tandem with thyroid hormones to enable the body to cope with an increase in metabolic rate (73–76). However, in the absence of a standard protocol, patients are discussing these treatment variations in a plethora of online discussion forums (149).

Several possible mechanisms have been proposed to explain positive outcomes of thyroid hormone supplementation in euthyroid patients (see section: Addressing low thyroid hormone *function*). In addition to their effects on mitochondrial activity, O&NS balance, immune function and neural activity, thyroid hormones stimulate ACTH secretion by the pituitary (127, 141, 150, 151). Supplementation with thyroid hormones might thus be directly relieving the central suppression of endocrine axes in *prolonged* critical illness and ME/CFS (see section: Reactivation of the adreno-cortical axis).

In this context it is necessary to mention that thyroid gland diseases (i.e., hypothyroidism, thyroiditis, and thyroid nodules) are frequently comorbid with ME/CFS (152–154). The standard medical practice in the case of underperforming thyroid glands is to prescribe levothyroxine (T4) with the aim of normalizing TSH levels. However, some researchers (in addition to the practitioners cited above) are increasingly warning that low thyroid hormone *function* in target cells (and associated symptoms) can persist despite the normalization of TSH levels with T4 treatment because of dysfunctions in the tissue-specific conversion and cellular uptake of thyroid hormones (132, 155–163)—particularly in the context of illness (as described above) or genetic polymorphism in thyroid hormone deiodinases and transporters (164–166). Consequently, these authors (and the many thyroid patient advocate groups around the world) promote treatments with T3 or T4/T3 combinations to ensure the adequate availability of the “active” thyroid hormone (T3) for target cells.

In summary, the use of thyroid hormones has been trialed for both *prolonged* critical illness and ME/CFS euthyroid patient groups. Although thyroid hormone supplementation suppresses endogenous thyroid hormone production via negative feed-back loops, their benefits may yet justify their use in the context of low thyroid hormone *function*. Positive results from early trials in ME/CFS and fibromyalgia—as well as anecdotal evidence described by ME/CFS practitioners and patients in numerous books—indicate that treatment for ME/CFS with thyroid hormone supplementation merits further investigation. The form of thyroid hormone supplementation (T3 vs. T4) is increasingly recognized as a determining factor in treatment success.

#### Treatments With Peripheral Hormone Combinations

##### In Prolonged Critical Illness

There is evidence that supplementation with a combination of peripheral hormones may lead to better survival and recovery in critical illness—compared to single hormone therapies. For example, the addition of GH and/or IGF-1 has been shown to mitigate the catabolic effects (e.g., protein wasting and osteoporosis) linked to high dose glucocorticoid treatments (167, 168). Moreover, critical care researchers have described the effects of hormones across endocrine axes (2, 169)—such as the stimulatory effect of GH on the T4 to T3 conversion (88) and the hypothalamic suppression of thyroid hormone production by high cortisol levels (11)—increasing the complexity in mitigating the effects of suppressed endocrine axes with peripheral hormones.

##### In ME/CFS

Similarly, ME/CFS practitioners treating patients with peripheral hormones typically prescribe a combination of hormones, including thyroid hormones, adrenal hormones (e.g., hydrocortisone, prednisone, pregnenolone, DHEA, and fludrocortisone) and even gonadal hormones (e.g., testosterone, progesterone and estradiol) (67, 73–78). Generally, the justification provided for this approach is the complementarity in the function of various hormones. The interactions between endocrine axes—such as the inhibitory effect of glucocorticoid on GH release (101)—described in ME/CFS and related studies also contribute to the rationale for peripheral hormone combination therapies (170–173).

In summary, trials using combinations of peripheral hormones often report beneficial outcomes in *prolonged* critical illness and ME/CFS exceeding those of single hormone therapies. However, the complex interactions between hormones during such trials remain largely unexplored.

### Approach B: “Reactivation” of the Central Endocrine Glands

A number of critical illness researchers argue that instead of administering *peripheral* hormones, treatments to relieve suppressed endocrine axes in *prolonged* critical illness should target the central level of the endocrine axes (i.e., the pituitary and hypothalamus) (8, 19). The rationale is the following: (i) endocrine suppression during *prolonged* illness largely originates at the level of the hypothalamus (i.e., the hypothalamus is not sending the required signals to the pituitary); (ii) the pituitary and peripheral endocrine glands are in fact undamaged and could operate normally if given the signals by the hypothalamus; and (iii) by targeting the central level, treatments can avoid altering the rest of the endocrine axes—specifically, the negative feedback loops and adaptive peripheral metabolism of hormones remain intact, thus preventing the risk of toxic over-dosages. Proponents of treatments targeting the hypothalamus and/or pituitary thus argue that these may be more effective and safer than administration of the peripheral hormones. In the next paragraphs we describe trials to reactivate central endocrine glands from the field of critical medicine, and relate these to similar trials to improve outcomes in ME/CFS and fibromyalgia.

#### Reactivation of the Adreno-Cortical Axis (HPA Axis)

##### In Prolonged Critical Illness

**Figure 1:**
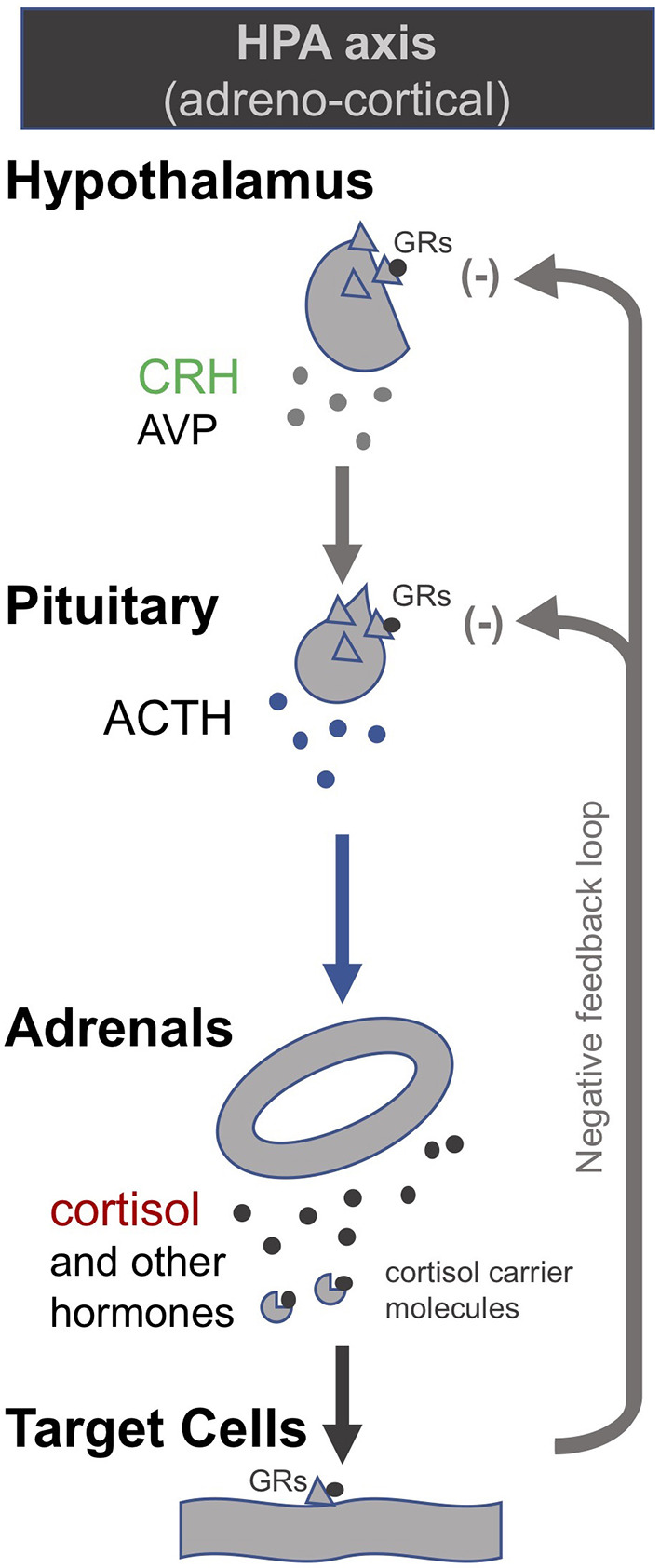
The adreno-cortical axis (HPA axis) [modified from (34)].

In order to correct the suppressed HPA axes in *prolonged* critical illness, researchers have suggested stimulating the pituitary with corticotropin-releasing hormone (CRH) (174). CRH is the tropic hormone by which the hypothalamus signals to the pituitary to produce ACTH, which in turn signals to the adrenal glands to produce the various peripheral adrenal hormones (Figure 1). Researchers have shown that high levels of *free* cortisol during the *acute* phase of critical illness driven by peripheral mechanisms (specifically, a decrease in the abundance and affinity of the cortisol carrier molecules in circulation, and a slowing of cortisol breakdown in the liver and kidney) lead to a suppression of the release of CRH in the case of *prolonged* critically ill patients even after cortisol levels have returned to normal. Pro-inflammatory cytokines and O&NS likely play a leading role (8, 10, 18, 19, 174). Researchers liken this to the suppression of the HPA axis in patients on long-term glucocorticoid treatment. *Prolonged* critically ill patients (19, 175) and patients on long-term glucocorticoid treatment (176, 177) also both experience adrenal atrophy; it is the lack of pulsatile ACTH stimulation of the adrenal glands that results in their atrophy (178). Consequently, these critical illness researchers argue that, akin to patients that are being withdrawn from long-term glucocorticoid treatment, *prolonged* critically ill patients also require the reactivation of pituitary secretions. In the case of long-term glucocorticoid treatment, slowly weaning patients off glucocorticoids permits the pituitary to secrete ACTH which in turn promotes the regeneration of adrenals; this can take anywhere from 6 to 12 months (176, 177). In the case of *prolonged* critical illness, some researchers propose the administration of CRH may be necessary to reactivate ACTH synthesis by the pituitary (19, 174). Initial trials on *prolonged* critically ill patients show that the pituitary responds as expected to stimulation with CRH (174).

##### In ME/CFS

Echoing the findings in *prolonged* critical illness, researchers have found evidence suggesting that hypoadrenalism in ME/CFS is caused by a central deficiency of CRH (50, 52, 55, 179–181). Moreover, significant adrenal atrophy has been documented in ME/CFS patients (182) and is also surmised to be present in fibromyalgia patients (98). Researchers have proposed a “bi-stability model” that serves to explain the persistence of a suppressed HPA axis in ME/CFS (183–189)—summarized in our earlier publication (34). These researchers suggest various interventions to move patients from a “low-cortisol” to a “normal-cortisol” HPA axis steady-state. In the words of one team: “a well-directed push given at the right moment may encourage the axis to reset under its own volition” (184). Some suggest artificially dropping cortisol levels even further than they already are in ME/CFS patients could be such a push (184). Models show that, given the HPA axis' negative feedback loop, this is expected to spur an increase in ACTH secretion and, as a result, the HPA axis will naturally progress to the “normal-cortisol” HPA axis steady-state, allowing treatment to be discontinued. Similarly, others have modeled the effect of blocking the glucocorticoid receptors to reset the HPA axis in ME/CFS patients (185). They argue that this intervention renders the “low-cortisol” steady-state unstable and thereby favors a return to the “normal-cortisol” steady-state. Finally, other researchers who have included immune system aspects in their model calculated that an initial inhibition of Th1 inflammatory cytokines (Th1Cyt), followed by a subsequent inhibition of glucocorticoid receptor function, would allow a robust return to a “normal-cortisol” steady-state in patients suffering from Gulf War Illness (186). However, given that a chronic suppressed HPA axis leads to adrenal atrophy—the result of prolonged under-stimulation of the adrenal glands by ACTH (178)—a switch to a “normal-cortisol” HPA-axis steady-state is necessarily a gradual process paced by the capacity for adrenal regeneration (190).

In this context it is necessary to mention that some researchers have administered CRH to ME/CFS patients—not in order to assess therapeutic potential, but in order to evaluate HPA-axis dysfunction (51, 52, 56, 62, 191, 192). Several studies found that the response to CRH injection was blunted in ME/CFS patients compared to controls (i.e., ensuing cortisol or ACTH production were lower than in controls) (51, 56, 62), but this was not the case when CRH was combined with desmopressin (a synthetic analog of arginine vasopressin, AVP) which acts synergistically with CRH on the pituitary to stimulate ACTH secretion (52). Similar tests performed with fibromyalgia patients found an exaggerated ACTH response, but blunted cortisol response to CRH injection (98, 101, 170, 193). Finally, ME/CFS patients were found to have “a blunted serum DHEA response curve to i.v. ACTH injection” (54) and lower cortisol production (194). These studies generally discuss possible mechanisms for the blunted HPA axis response, including elevated levels of CRH-binding protein, enhanced sensitivity to the negative feedback of glucocorticoids (i.e., a higher abundance of glucocorticoids receptors at central level), secondary adrenal atrophy, etc. However, the therapeutic potential of CRH (or other pituitary secretagogues) to relieve the suppressed HPA axis in ME/CFS has generally not been considered.

In summary, researchers in both *prolonged* critical illness and ME/CFS have sought the reactivation of the HPA axis. Researchers from the field of critical medicine suggest the use of pituitary secretagogues; ME/CFS researchers suggest that an endogenous “push” could serve to revert the HPA axis to a “normal-cortisol” steady-state. Adrenal atrophy evidenced in both conditions—a result of chronic under-stimulation of adrenals by ACTH—implies that reversing hypoadrenalism in both conditions is a gradual process.

#### Reactivation of the Somatotropic (HPS) and Thyrotropic (HPT) Axes

##### In Prolonged Critical Illness

**Figure 2:**
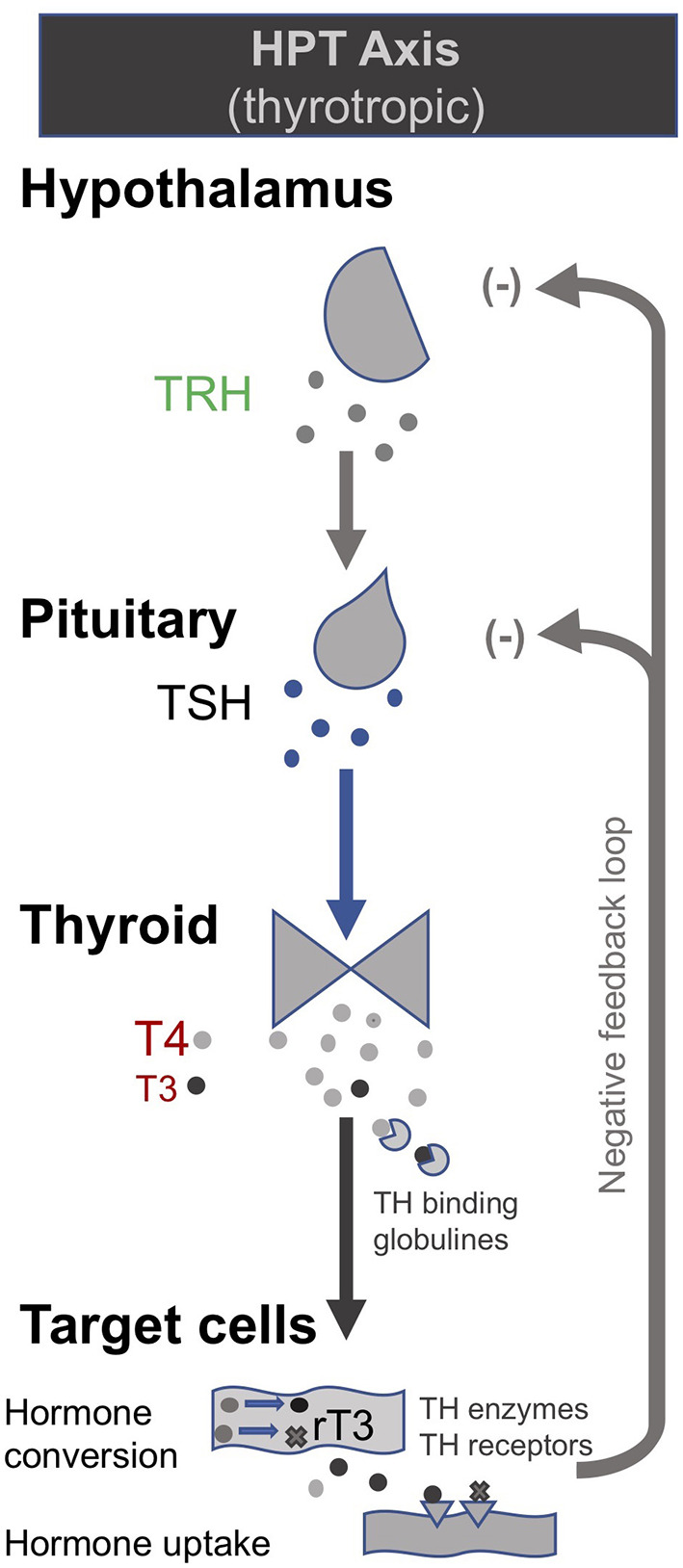
The somatotropic axis (HPS axis) [modified from (34)].

**Figure 3:**
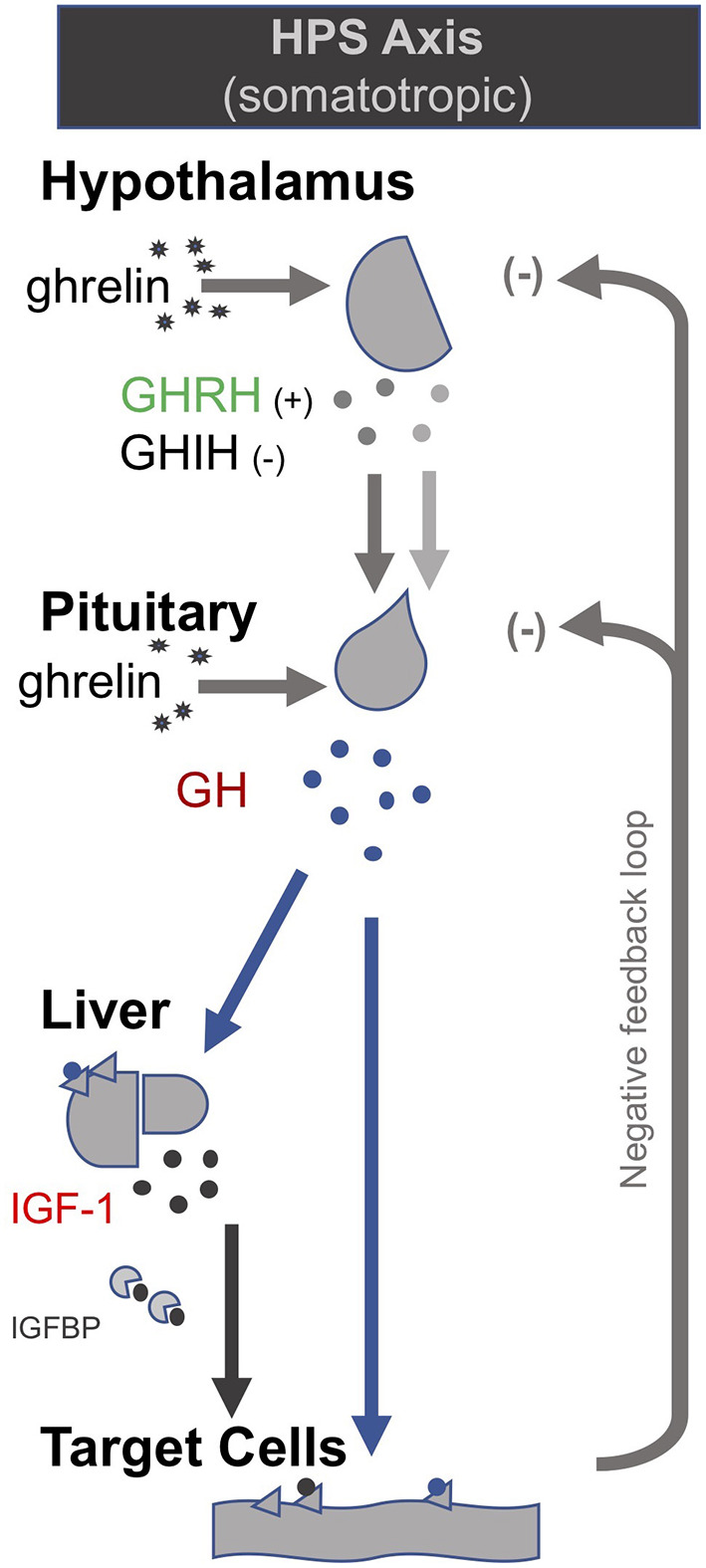
The thyrotropic (HPT axis) [modified from (34)].

In the late 1990s van den Berghe and her team administered secretagogues that stimulate the pituitary to critically ill patients who had been in ICUs for several weeks, thereby supplementing signals produced by the hypothalamus (195, 196). Specifically, they administered thyrotropin-releasing hormones (TRH) and GH-releasing hormone (GHRH). These two secretagogues, respectively, target the hypothalamic–pituitary–thyroid (HPT) and the hypothalamic–pituitary–somatotropic (HPS) axes. TRH stimulates the pituitary to produce thyroid stimulating hormone (TSH), which in turn signals to the thyroid gland to produce thyroid hormones (197) (Figure 2). GHRH stimulates the pituitary to produce GH which in turn stimulates the production of IGF-1 mostly by the liver (in addition to direct effects of GH on some tissues). Nearly all of the IGF-1 hormones in the plasma are bound to IGF-binding proteins (IGFBP) (198) (Figure 3). As an alternative to GHRH the researchers also trialed the use of GHRP-2, an artificial ghrelin-like peptide that also stimulates the pituitary to produce GH.

These trials showed that each of these secretagogues can reactivate the secretion by the pituitary for the relevant endocrine axis, while keeping the negative feedback loops on the pituitary intact, thus preventing overstimulation of the endocrine glands. Specifically, the administration of GHRH or GHRP-2 reactivated the pulsatile secretion of GH by the pituitary, and the plasma concentrations of IGF-1 and IGFBP-3 increased [Interestingly, GHRP-2 had a much stronger effect than GHRH, suggesting that the inactivity of ghrelin likely plays a key role in *prolonged* critical illness (8)]. Similarly, when the team administered TRH, the pulsatile secretion of TSH by the pituitary was reactivated, and the plasma concentrations of the peripheral hormones T4 and T3 increased. However, in the latter case reverse T3 (rT3)—an *inactivated* form of thyroid hormone—also increased; this is considered problematic because rT3 contributes to low thyroid hormone *function* (see section: Addressing low thyroid hormone *function*).

Crucially, the team showed that when *prolonged* critically ill patients were treated with a combination of the secretagogues to normalize GH and TSH secretion by the pituitary (i.e., GHRH or GHRP-2 in combination with TRH), plasma rT3 concentrations did *not* increase. This is likely because GH can deactivate the D3 enzyme which converts T4 into rT3 (88), and suggests that the normalization of the HPS axis is necessary to inhibit the production of rT3. Moreover, the administration of both secretagogues immediately inhibited *catabolism* (i.e., tissue break-down) and promoted *anabolism* (i.e., tissue building), thus halting the muscle and fat wasting of patients with *prolonged* critical illness. This has important both short- and long-term clinical consequences since the impaired neuromuscular function is considered to be the factor which most strongly correlates with the severely impaired quality of life in critical illness survivors several years after hospital discharge (199–202).

The treatments were only administered for 5 days for experimental purposes, and benefits ended a few days after the infusions were discontinued. Nonetheless, they demonstrated the possibility of reactivating suppressed endocrine axes in *prolonged* critical illness with secretagogues targeting the pituitary, as well as positive metabolic outcomes.

##### In ME/CFS

There are no comparable trials to reactivate the HPS and HPT axes in ME/CFS. One study did administer GHRH to ME/CFS patients but only for the purpose of testing the function of the HPS axis; they found GH responses to stimulation with GHRH were no different in patients and controls, and also found no GH deficiency in ME/CFS (203). (These findings are consistent with our hypothesis that *pulsatile* pituitary GH secretions are suppressed in ME/CFS). There have, however, been attempts to reactivate pituitary GH secretions in fibromyalgia patients. Recognizing that the secretion of GH by the pituitary is controlled by both *stimulating* and *inhibiting* signals from the hypothalamus [i.e., both GH-releasing hormone (GHRH) and GH-inhibiting hormone (GHIH)], researchers treated fibromyalgia patients with pyridostigmine, a drug that inactivates the inhibiting effect of GHIH. Pyridostigmine reversed the impaired GH response to exercise in fibromyalgia patients, indicating a correction of an otherwise depressed HPS axis (97). However, it did not improve fibromyalgia symptoms (204)—this appears consistent with van den Berghe et al.'s findings described above, whereby the metabolic effects of GH only occur in combination with adequate thyroid hormone *function*. Others have administered GHRH and arginine to fibromyalgia patients—not in order to assess therapeutic potential, but in order to evaluate HPS-axis dysfunction (100).

In summary, researchers have demonstrated that the reactivation of centrally suppressed HPS and HPT axes in *prolonged* critical illness with pituitary secretagogues leads to beneficial metabolic effects. The reactivation of the HPS and HPT axes in ME/CFS for therapeutic purposes remains largely unexplored.

#### Reactivation of a Combination of Endocrine Axes

##### In Prolonged Critical Illness

**Figure 4:**
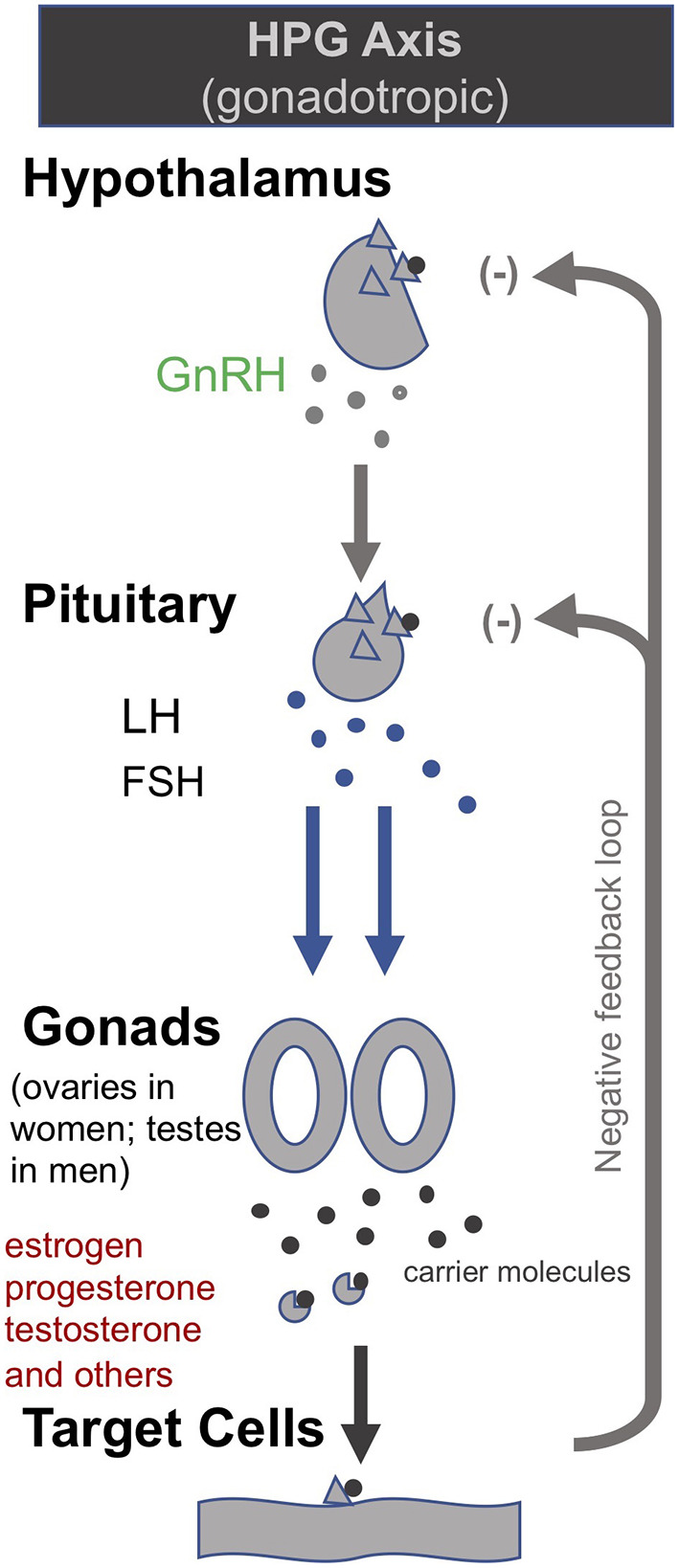
The gonadotropic (HPG axis) [modified from (34)].

Building on their earlier trials administering GHRH (or the synthetic peptide GHRP-2) and TRH in order to, respectively, stimulate the HPS and HPT axes as described above, van den Berghe et al. later also administered gonadotropin-releasing hormone (GnRH) to *prolonged* critically ill patients (205, 206). GnRH stimulates the pituitary to produce follicle-stimulating hormone (FSH) and luteinizing hormone (LH), which in turn stimulate the gonads to produce estrogen, progesterone and testosterone (c.f. Hypothalamic–pituitary–gonadal axis: “HPG axis”; Figure 4). They found that the positive metabolic effect was strongest with the combination of secretagogues stimulating all 3 axes (i.e., with GHRP-2, TRH and GnRH). The authors write: “coadministration of GHRP-2, TRH and GnRH reactivated the GH, TSH and LH axes in *prolonged* critically ill men and evoked beneficial metabolic effects which were absent with GHRP-2 infusion alone and only partially present with GHRP-2 + TRH. These data underline the importance of correcting the multiple hormonal deficits in patients with *prolonged* critical illness to counteract the hypercatabolic state” (206) (Table 1).

**Table 1:** Summary of the treatment trials to reactivate the pituitary in *prolonged* critical illness as described by van den Berghe et al. (195, 196, 205, 206).

**Target**	**Secretagogues used to stimulate the pituitary**	**Results in *prolonged* critical illness**
HPT Axis	**TRH** (which stimulates the pituitary to produce TSH, in turn stimulating the thyroid gland)	**Reactivation of the HPT Axis** Normalized TSH secretion by pituitary Normalized T4 and T3 levels *Increased RT3*
HPS Axis	**GHRP-2** (artificial ghrelin mimetic which stimulates the pituitary to produce GH)	**Reactivation of the HPS Axis** Normalized GH secretion by pituitary Normalized IGF-1 and IGFBP-3 levels
	**GHRH** (which stimulates the pituitary to produce GH)	**Reactivation of the HPS Axis** Lower pituitary reactivation response than with GHRP-2
Combination HPS + HPT Axes	**GHRP-2 + TRH**	**Reactivation of the HPS and HPT Axes** Normalized GH secretion by pituitary Normalized IGF-1 and IGFBP-3 levels Normalized TSH secretion by pituitary Normalized T4 and T3 levels **RT3 levels do not increase!** ***-*****> Inhibit** ***catabolism*** **and promote** ***anabolism***
Combination HPS + HPT + HPG Axes	**GHRP-2 + TRH + GnRH** (GnRH stimulates the pituitary to produce LH and FSH; trialed with men)	**Reactivation of the HPS, HPT and HPG Axes** As above and also normalized LH secretion by the pituitary **-> Strongest beneficial** ***metabolic*** **effect**

##### In ME/CFS

We are not aware of any efforts to simultaneously reactivate the pituitary secretions for a combination of endocrine axes in ME/CFS or fibromyalgia in order to improve metabolic outcomes. Researchers have injected fibromyalgia patients simultaneously with CRH, TRH, GHRH as well as GnRH—however, this was not done in order to assess the therapeutic potential of secretagogues but to study the patients' endocrine dysfunctions (170). They found that the injection of the four releasing-hormones led to an “exaggerated” ACTH secretion compared to controls; this was not the case when CRH was administered alone [see above (101)]. They also highlighted the inhibitory effects of CRH on TSH and GH secretion. These results appear consistent with van den Berghe's findings that a combination of secretagogues most effectively improve metabolic outcomes during *prolonged* critical illness.

In summary, researchers have demonstrated that the concurrent reactivation of HPS, HPT and HPG axes with pituitary secretagogues in *prolonged* critical illness leads to greater beneficial metabolic effects than reactivation of just one or two of the axes. The concurrent reactivation of endocrine axes in ME/CFS for therapeutic purposes remains largely unexplored.

### Intermediate Conclusions

There are important similarities in the efforts to correct endocrine suppression in *prolonged* critical illness and ME/CFS (Table 2). For both conditions, researchers have trialed supplementation with peripheral hormones, including hydrocortisone, GH and IGF-1, and thyroid hormone (including T4 and T3). Evidence exists of some benefits from these treatments for patients suffering from either illness, particularly when given in combination. However, difficulties with finding optimal dosing and risks of causing harm to patients have contributed to controversies and limited their application for both *prolonged* critical illness and ME/CFS. Moreover, administration of these peripheral hormones exasperates central suppression of the respective endocrine axes.

**Table 2:** Summary of treatments proposed and trialed to correct for suppressed endocrine axes in critical illness, and ME/CFS and fibromyalgia.

**Target**	**Approach A: Treatments with peripheral hormones**	**Approach B: “Reactivation” of the central endocrine glands**
HPA Axis (adrenals)	***Prolonged*** **critical illness:** High dose hydrocortisone (5, 48)	***Prolonged*** **critical illness:** Administration of CRH to stimulate pituitary ACTH secretion (proposed) (174)
	**ME/CFS or fibromyalgia:** Low dose hydrocortisone [see reviews (67, 68)], fludrocortisone (79–84), DHEA (85), and pregnenolone (proposed) (86).	**ME/CFS or fibromyalgia:** - Suppress cortisol levels to reactivate ACTH secretion (modeled) (184) - Blocking of central glucocorticoids receptors (GRs) (modeled) (185) - Inhibition of Th1 cytokines followed by inhibition of GRs (modeled for Gulf War Illness) (186)
HPS Axis (growth hormone)	***Prolonged*** **critical illness:** Supplementation with GH and IGF-1 [see reviews (87, 88, 90, 91)]	***Prolonged*** **critical illness:** Administration of GHRH and GHRP-2 to reactivate pituitary secretion of GH (195, 196)
	**ME/CFS or fibromyalgia:** Supplementation with GH and IGF-1 (94, 102–105)	**ME/CFS or fibromyalgia:** Drug to inactivate GH inhibiting hormone (GHIH) (97, 204)
HPT Axis (thyroid)	***Prolonged*** **critical illness:** Supplementation w/thyroid hormones [see reviews (116, 117)]	***Prolonged*** **critical illness:** Administration of TRH to reactivate pituitary secretion of TSH (195)
	**ME/CFS or fibromyalgia:** Supplementation w/thyroid hormones (natural desiccated thyroid, T4, T3) (135–140) Anecdotal: (67, 73–78, 127, 141–145, 147, 148)	**ME/CFS or fibromyalgia:** *none?*
HPG Axis (gonads)	**Prolonged critical illness:** anabolic steroid (e.g., testosterone) (207)	***Prolonged*** **critical illness:** Administration of GnRH to stimulate pituitary release of LH (in men) (206)
	**ME/CFS or fibromyalgia:** as part of combined treatments (see below)	**ME/CFS or fibromyalgia:** *none?*
Combination of axes	***Prolonged*** **critical illness:** GH and IGF-1 in addition to hydrocortisone (167, 168)	***Prolonged*** **critical illness:** TRH + GHRP-2 + GnRH (see Table 1. above) (206)
	**ME/CFS or fibromyalgia:** Thyroid hormone + adrenal hormones (+ gonadal hormones) (67, 73–78)	**ME/CFS or fibromyalgia:** *none?*

Researchers of *prolonged* critical illness have also trialed to reactivate endocrine axes at the central level. Unlike treatments with peripheral hormones, such an approach has the benefit that it addresses the central suppression of endocrine axes directly, and that peripheral endocrine glands are stimulated—this is particularly important for the adrenal glands which without stimulation by ACTH go into atrophy. Given that negative feed-back loops and peripheral hormone metabolism remain intact, proponents argue that the approach is safer and more effective for restoring normal endocrine function. Trials on *prolonged* critical illness patients to reactivate pituitary secretions with secretagogues including CRH, GHRH (or the synthetic GHRP-2), and GnRH have had important initial successes not only in restoring *pulsatile* pituitary secretions of ACTH, GH, and LH, respectively, but also in achieving positive metabolic effects when administered in combination. Moreover, these trials also offer revelations about the interactions between the endocrine axes—including the insight that deleterious production of rT3 can be mitigated through the simultaneous reactivation of the HPT and HPS axes. These lessons-learned from the field of critical illness can complement and inform ME/CFS research. ME/CFS researchers, in turn, have proposed interventions to reactivate the HPA axis based on a “bi-stability model” with two HPA axis steady-states. However, the simultaneous reactivation of the endocrine axes in ME/CFS remains unexplored. The potential of treatments with pituitary secretagogue to correct central endocrine axes suppression—and to enable the reversal of secondary adrenal atrophy—should be assessed for ME/CFS.

## Interruption of the “Vicious Circle”

Critical illness researchers have proposed a model for how *prolonged* critical illness is perpetuated by reciprocal relationships between inflammation (notably pro-inflammatory cytokines), O&NS and reduced thyroid hormone *function* (13, 16). Simplified, this model suggests that (i) cytokines depress thyroid hormone *function*; (ii) low thyroid hormone *function* contributes to O&NS; and (iii) O&NS in turn stimulates the production of pro-inflammatory cytokines, thereby completing a “vicious circle.” Moreover, reciprocal relationships between these elements and the suppressed endocrine axes [e.g., pro-inflammatory cytokines suppress ACTH release (9, 10); weakened adrenals permit excessive inflammatory responses] contribute to perpetuate a hypometabolic and inflammatory state (208), and thus help to explain why some critically ill patients fail to recover. Crucially, the same elements of such a “vicious circle” have also been documented in ME/CFS (134, 209–216)—as described in our earlier publication (34).

Treatment trials to improve survival and recovery from *prolonged* critical illness have often targeted one of the elements of this “vicious circle.” In the following paragraphs we provide an overview of these various treatment trials using the “vicious circle” as a framework. We also relate analogous experimental treatments for ME/CFS and fibromyalgia in order to highlight the similarities in the quests to cure both *prolonged* critical illness and ME/CFS, and to derive lessons for solving ME/CFS.

### Addressing Low Thyroid Hormone *Function*

#### In Prolonged Critical Illness

As described above (section: Treatments with thyroid hormones), clinicians began, as early as the 1970s, to suggest thyroid hormone supplementation for critically ill patients. The rationale was to correct what some considered a *maladaptive* hypo-metabolic state which was preventing recovery following *severe* infection or injury (106–108). Again, this approach remains controversial (111–115). Interestingly, there has been little research into pharmacological agents to correct the peripheral mechanisms, which to a large extent underpin the low thyroid hormone *function* during critical illness: i.e., the alterations in cellular thyroid hormone transporters, receptors, and (most crucially) deiodinases that convert thyroid hormones into their “active” and “inactivated” forms. Targeting these deiodinases could theoretically be an avenue for alleviating low thyroid hormone *function* during *prolonged* critical illness (217).

#### In ME/CFS

As described above, there are accounts of positive effects of thyroid hormone supplementation to address low thyroid hormone *function* in euthyroid ME/CFS and fibromyalgia (67, 73–78, 127, 135–145, 147, 148). Proponents generally believe that thyroid hormone supplementation serves to compensate for dysfunctions in the conversion of thyroid hormones (from “inactive” to “active” forms) and/or uptake at cellular level (139, 218–223), notably associated with inflammation in ME/CFS or fibromyalgia (224, 225).

However, the mechanisms by which positive metabolic effects were achieved with thyroid supplementation in ME/CFS or fibromyalgia are not entirely clear. Rat models show that T3 and T2 thyroid hormone injections can repair mitochondrial DNA (mtDNA) damage resulting from oxidative stress (226). In cells of patients with mtDNA mutations, administration of T3 led to a *reduction* in reactive oxygen species (ROS) production (i.e., oxidative stress) and a *reduction* in cytoplasmic Ca2+ (allowing for cellular signaling/regulation of enzymes and proteins). Moreover, cytochrome c oxidase activity (involved in ATP production) and ATP levels were increased. T3 also restored the mitochondrial membrane potential, complex V activity, and levels of manganese superoxide dismutase (an essential mitochondrial antioxidant enzyme). The authors conclude that “the results suggest that T3 acts to reduce cellular oxidative stress, which may help attenuate ROS-mediated damage, along with improving mitochondrial function and energy status in cells with mtDNA defects” (227). In theory, T3 supplementation could have similar impacts on relieving O&NS and improving mitochondrial function in *prolonged* critical illness and ME/CFS.

Moreover, T3 (but not T4) administration also stimulated Na-K-ATPase activity in rat models through non-genomic pathways (the activity of this enzyme is critical for maintaining cellular ion gradients) (228). Others have found that T3 and T4 supplementation selectively affect GABA-evoked neurotransmission in rat models thus possibly producing profound alterations in brain activity (229, 230). In addition, it has been shown that thyroid hormones also have a neuroprotective effect (231) and regulate neurotransmission (232, 233). Moreover, clinical manipulation of thyroid hormone levels also modulates immune functions (234–237). Yet others have shown that T3 treatment of human cells caused decreased viral replication (238). Finally, as mentioned above, thyroid hormones also have a stimulatory effect on the HPA axis.

In summary, there is a history of clinicians in both the fields of critical illness and ME/CFS advocating for the use of thyroid hormones supplementation in euthyroid patients. Thyroid hormones affect mitochondrial activity, O&NS balance, immune function, neuroactivity, and stimulate ACTH secretion. The mechanisms by which supplementation with thyroid hormone can promote recovery from *prolonged* critical illness and ME/CFS require further investigations. Again, the form of thyroid hormone (T4, T3, or T2) appears to be a determining factor in physiological effects of treatments. The use of pharmacological agents to correct dysfunctions in the peripheral pathways of thyroid hormones remains unexplored in both illnesses.

### Addressing Oxidative and Nitrosative Stress

#### In Prolonged Critical Illness

There have been a few trials to restore oxidative balance during critical illness, often with the aim of improving thyroid hormone *function* (i.e., relieving NTIS). In one case, researchers found that treating patients of acute myocardial infarction with n-acetyl-cysteine (NAC)—a precursor to the antioxidant glutathione (GSH)—could virtually eliminate the decrease in serum T3 levels and prevent the increase in serum rT3 which are characteristic of NTIS (239). They propose that supplementation with NAC relieves the competition for GSH between the thyroid hormone deiodinase and antioxidant enzymes, which would otherwise negatively affect thyroid hormone conversion and enable O&NS. Likewise, controlled experiments showed that administration of sodium selenite on human cells reduces cytokine-induced oxidative stress (240), and supplementation with selenium is associated with modest normalization of thyroid hormones during critical illness (241). Thus, *in-vivo* supplementation might similarly relieve competition for selenium required in the production of both thyroid hormone deiodinase and antioxidant enzymes.

Furthermore, cytokine-activated oxidative stress induced post-translational modifications and loss of the molecular motor protein myosin are important pathophysiological mechanisms underlying the severe muscle dysfunction and muscle wasting associated with the critical illness myopathy (CIM) and the ventilator induced diaphragm dysfunction (VIDD) observed in both experimental and clinical ICU studies in response to long-term mechanical ventilation and immobilization (242–246). Administration of the chaperone co-inducer BGP-15 upregulates Heat Shock Proteins (HSPs) and mitigates the muscle dysfunction associated with CIM and VIDD (247–249). This is consistent with HSP protection of muscle cells against the damaging effects of reactive oxygen species during exercise (250). Besides upregulating HSPs, BGP-15 also acts as a membrane stabilizer, protects mitochondria, and has anti-inflammatory effects (251). The anti-inflammatory effects are of specific interest since activation of the JAK/STAT signaling pathway has been reported in respiratory and limb muscles in both experimental and clinical ICU studies (252, 253). The JAK/STAT pathway is a signaling pathway for a wide range of cytokines and growth factors and its activation is a common feature of muscle wasting induced by the cytokine IL-6 (254). Previous anti-inflammatory interventions with BGP-15, the JAK1/2/STAT3 inhibitor Ruxolitinib, and the prednisolone analog Vamrolone have all shown positive effects on limb and respiratory structure/function (252, 253, 255).

#### In ME/CFS

Addressing antioxidant status is a common approach of some ME/CFS practitioners (73, 74, 211), notably with the aim of preventing mitochondrial damage (256). Pall—who described a “vicious cycle” between inflammation and oxidative stress in ME/CFS more than a decade ago (211, 257)—developed a treatment protocol based on a variety of antioxidants and anti-inflammatory agents. Moreover, placebo-controlled studies have shown that CoQ10—an important antioxidant in mitochondria—is beneficial to ME/CFS patients when provided in combination with the coenzyme NADH (258, 259). Following early positive results, a study is currently ongoing to determine the efficacy of NAC in neuroprotection against oxidative stress in ME/CFS symptoms (260). Furthermore, a placebo-controlled trial of the herbal medicine myelophil—with antioxidant and immunomodulatory properties—has had promising results in alleviating ME/CFS symptoms; the benefits may also derive from its modulatory effects on the HPA axis (68, 261).

Finally, akin to critical illness, it has been suggested (but not yet trialed) to incorporate the upregulation of HSP into future treatments for ME/CFS (262). Studies have shown that ME/CFS is also characterized by impaired HSP production (263) which—combined with O&NS and low-grade inflammation—could explain muscle dysfunction and exercise intolerance (264, 265).

In summary, there have been a few trials to mitigate O&NS in both *prolonged* critical illness and ME/CFS patients. This includes the use of various antioxidants and mitochondrial supports to rebalance oxidative stress, and the use of HSP to lessen the negative effects of O&NS. There is evidence of some beneficial results, but effects may be insufficient to interrupt detrimental and possibly self-perpetuating mechanisms.

### Addressing the Production of Pro-inflammatory Cytokines and Inflammation

#### In Prolonged Critical Illness

As described above, clinical practitioners regularly seek to manage inflammation during critical illness—particularly in the event of sepsis (see section: Treatments with glucocorticoids). Considering the relationship between pro-inflammatory cytokines and thyroid hormone *function* (9–15, 266), some researchers have tried unsuccessfully to cure NTIS (i.e., restore normal thyroid hormone *function*) by blocking IL-1 cytokine receptors (267). The unsuccessful result is perhaps not surprising, given that “cytokines are related to each other in a very complex network, and regulate positively or negatively the expression of other cytokines; it is, therefore, difficult to imagine how to interrupt this interplay and cascade of events” (268). Critical care researchers also debate using antivirals (269, 270) to treat viral reactivation observed in ICU patients (271–273).

Related to inflammation, a group of researchers has suggested inhibiting the kynurenine pathways during critical illness (274). In conditions of inflammation the indoleamine 2,3-dioxygenase (IDO) (which metabolizes tryptophan into kynurenine) is upregulated, and the kynurenine pathway preferentially produces neurotoxic metabolites (such as quinolinic acid) (274, 275). Increased kynurenine plasma levels thus precede the development and persistence of sepsis in critically ill patients (276, 277), and is associated with lower survival in ICU patients (274). Moreover, elevated kynurenic acid (also a metabolite of kynurenine) is associated with myelin damage leading to neuronal and cognitive dysfunction in critical illness (278). Based on the therapeutic literature from other diseases (275, 279–282), the critical illness researchers suggest inhibiting the IDO enzyme in order to curtail the production of neurotoxic kynurenine pathways metabolites.

#### In ME/CFS

Echoing the approaches in critical care, practitioners and researchers have also long sought to manage inflammation in ME/CFS (29, 73, 211). Trials include the use of Nexavir (Kutapressin) to reduce inflammation (283) as well as the administration of Low Dose Naltrexone (LDN) (284, 285) and IL-1 receptor antagonist (anakinra) (286) to reduce pro-inflammatory cytokines. There is evidence of some positive benefits from anti-inflammatory treatments for ME/CFS patients, notably with LDN.

Researchers have also trialed treatments which could have an indirect effect on inflammation. These include the use of cyclophosphamide (287) and monoclonal antibodies (rituximab) (288) which suppress the immune system, as well as immune adsorption (IgG depletion) and plasmapheresis (filtration of blood plasma) to reduce antibodies (289). Others have conversely tried to modulate the immune system, including through the use of toll-like receptor 3 (TLR3) agonists (rintatolimod/Ampligen) (290, 291), immune-stimulants such as Imunovir (292), and intravenous gamma globulin (293). There have also been trials targeting infections directly, including with antivirals (acyclovir and valganciclovir) (294–297) for chronic viral infections in ME/CFS patients (298–300). There is evidence of some positive benefits, at least for a subset of patients, from some of these studies, but results remain largely inconclusive or subject to controversies; readers are referred to reviews for details (29–32).

Finally, the modulation of kynurenine pathways has also been suggested as a therapeutic avenue for ME/CFS (301, 302). However, in contrast to the approach suggested by critical illness researchers, the initial emphasis of ME/CFS research is on enabling rather than inhibiting the activity of the IDO enzyme. Given that the downstream kynurenine pathways produce both beneficial and neurotoxic kynurenine metabolites (275)—and neurotoxic metabolites are preferentially produced in conditions of inflammation (274)—pharmacological agents that target specific enzymes of the kynurenine pathways may be required in order to maintain a beneficial balance of the various metabolites (303). A recent study demonstrated the safety of L-kynurenine supplementation in healthy volunteers (304); the impacts on ill patients will need to be further investigated.

In summary, many efforts in both *prolonged* critical illness and ME/CFS have focused on mitigating inflammatory processes and/or modulating the immune system in patients. There is evidence of some beneficial results from some of these studies, but effects appear insufficient to interrupt detrimental and possibly self-perpetuating mechanisms. Antivirals have also been trialed for both *prolonged* critical illness and ME/CFS (viral reactivation has been documented in both illnesses). In both fields there has also recently been discussion of modulating the kynurenine pathways to rebalance beneficial and neurotoxic metabolites.

### Intermediate Conclusions

**Figure 5:**
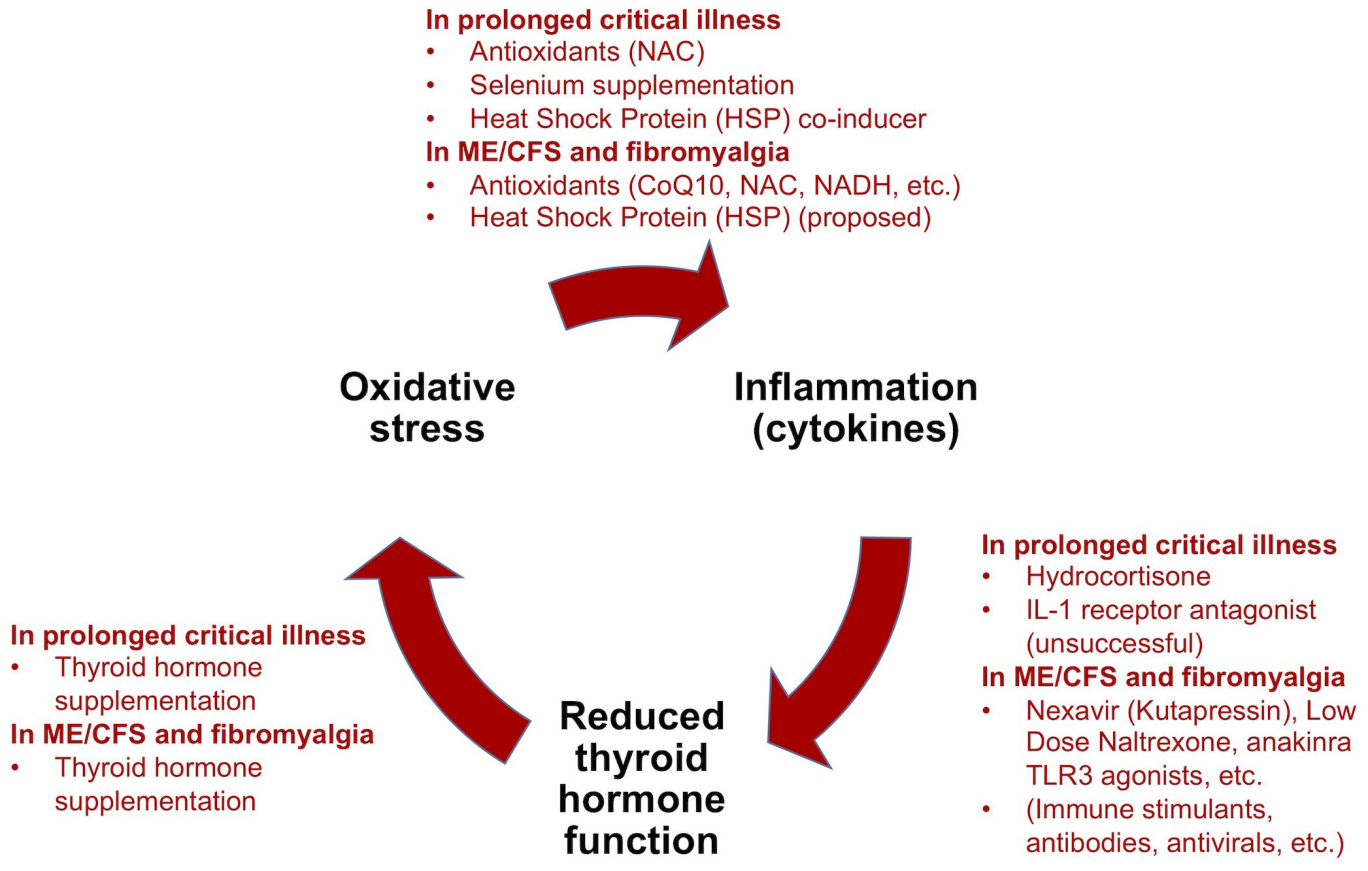
Summary of treatment trials for *prolonged* critical illness using the “vicious circle” model as a framework, and analogous experimental treatments for ME/CFS and fibromyalgia [modified from (34)].

Treatment trials for *prolonged* critical illness and ME/CFS (or fibromyalgia) have both independently targeted low thyroid hormone *function*, O&NS, and pro-inflammatory cytokines and inflammation (Figure 5). Evidence exists for some benefits from these treatments in both conditions, but treatment trials have generally been limited in their scope, number and impact. Consequently, results have not translated into standard practices in either field. Further studies are required to fill gaps in the understanding of the physiological mechanisms behind some positive results, such as in the case of supplementation with thyroid hormones in both *prolonged* critical illness and ME/CFS.

## Additional Considerations

As mentioned in the introduction, we previously advanced the hypothesis that maladaptive mechanisms that prevent recovery in *prolonged* critical illness may also underlie ME/CFS—and propose that these mechanisms could underlie the perpetuation of illness in ME/CFS regardless of the nature of the peri-onset event (i.e., infection, stressful incident, exposure to environmental toxins or other) (34). Nonetheless, additional considerations must be taken into account when considering the relevance of treatment trials from critical care medicine for ME/CFS.

Firstly, the long disease duration in ME/CFS (relative to *prolonged* ICU patients) implies that dysfunctions that occur as a result of years of chronic disease must be considered as part of treatment approaches. Secondly, research suggests that dysfunctions change over time in ME/CFS patients (28) and that there may be ME/CFS disease sub-groups (305–307); this implies that disease subtyping is necessary in order to match treatments to the patients. Thirdly, given that ME/CFS patients—similar to *prolonged* critical illness patients—have multi-system dysfunctions (e.g., endocrine, immune, nervous system, etc.), the question of sequence and/or combination of treatments must be considered. Fourthly, the side-effects of treatments described in this paper may differ between *prolonged* critical illness and ME/CFS patients (and also across ME/CFS patients) not least because of differences in severity of illness and dysfunctions. Recognizing the differences in fragility and vulnerability to side-effects of patients, an assessment of the trade-offs of treatments is necessary. Finally, it is reasonable to hypothesize that patients would have to endure long treatment courses occurring sizable costs; yet the enormous total economic costs of ME/CFS [estimated at $36 to $51 billion annually in the USA (308)]—not to mention the high financial and emotional toll of the disease on the millions of patients and families worldwide—makes establishing and implementing effective treatments for ME/CFS a long overdue imperative.

In summary, ME/CFS and *prolonged* critical illness are not identical illnesses. Any discussion of the relevance of treatment trials from *prolonged* critical illness for ME/CFS should take into account the specificities of ME/CFS, notably the dysfunctions arising from years of illness, the progression of the disease over time, the possible existence of sub-groups of ME/CFS patients, and potential particular vulnerabilities to side-effects.

## Conclusion

There are significant parallels in the treatment trials to aid recovery in *prolonged* critical illness and ME/CFS. Treatments proposed or trialed for both of these conditions have targeted (a) the correction of suppressed endocrine axes, and/or (b) inflammation, O&NS, and/or low thyroid hormone *function*. Treatment trials to date have been limited in scope and number; both *prolonged* critical illness and ME/CFS remain unsolved conditions. Incidentally, the parallels in the treatment trials would support the hypothesis that maladaptive mechanisms that prevent recovery in *prolonged* critical illness could also underlie ME/CFS.

From the brief overview and comparison of these trials provided here, we can derive some preliminary lessons to be learned. Notably, the early successes to reactivate the *pulsatile* secretions of the pituitary in *prolonged* critically ill patients with pituitary secretagogues—and the resulting positive metabolic effects—would indicate that this also could be an important avenue for ME/CFS treatments. The simultaneous reactivation of suppressed endocrine axes so far remains unexplored in ME/CFS. Conversely, the findings from ME/CFS related to the dysfunctions at the cellular and mitochondrial level can likely provide important complementary insights to the understanding of critical illness. In addition, the positive impacts from thyroid hormone supplementation described in some of the trials for both conditions merit further investigation.

Chiefly, given the similarities described above, an exhaustive analysis of the treatments already tried for either *prolonged* critical illness or for ME/CFS could help identify potential approaches that could be immediately trialed for one or the other of these conditions. Moreover, active collaboration between critical illness and ME/CFS researchers to leverage their respective experiences could lead to improved outcomes for both conditions. More broadly—and given the similarities between *prolonged* critical illness, post-ICU syndrome, ME/CFS, fibromyalgia, and long-COVID—we suggest that collaborative efforts should be sought among the researcher community across these conditions in order to identify treatments mitigating the functional disability that they induce.

## Data Availability Statement

The original contributions presented in the study are included in the article/supplementary material, further inquiries can be directed to the corresponding author/s.

## Author Contributions

DS wrote the first draft of the manuscript. All authors contributed to manuscript revision, read, and approved the submitted version.

## Conflict of Interest

The authors declare that the research was conducted in the absence of any commercial or financial relationships that could be construed as a potential conflict of interest.

## Abbreviations

ACTH: Adrenocorticotropic hormone
AVP: Arginine vasopressin
CIM: Critical illness myopathy
CIRCI: Critical illness-related corticosteroid insufficiency
CRH: Corticotrophin-releasing hormone
GH: Growth hormone
GHIH: Growth hormone inhibiting hormone
GHRH: Growth hormone releasing hormone
GHRP-2: synthetic Growth hormone releasing peptide
GnRH: Gonadotropin-releasing hormone
GSH: Glutathione
HPA: hypothalamus-pituitary-adrenal axis: “Adreno-cortical axis”
HPG: Hypothalamic–pituitary–gonadal axis: “gonadotropic axis”
HPS: Hypothalamic-pituitary-somatotropic axis: “Somatotropic axis”
HPT: Hypothalamic-pituitary-thyroid: “Thyrotropic axis”
HSPs: Heat Shock Proteins
ICU: Intensive Care Unit
IDO: Indoleamine 2,3-dioxygenase
IGF-1: Insulin like growth hormone-1
IGFBP: Insulin like growth hormone binding proteins
JAK/STAT: Janus kinase—signal transducer and activator of transcription
LDN: Low Dose Naltrexone
LH: Luteinizing hormone
LHRH: Luteinizing hormone-releasing hormone
ME/CFS: Myalgic Encephalomyelitis/Chronic Fatigue Syndrome
mtDNA: Mitochondrial DNA
NAC: N-acetylcysteine
NADH: Nicotinamide adenine dinucleotide
NTIS: Non-thyroidal illness syndrome
O&NS: oxidative and nitrosative stress
PICS: Post-intensive care syndrome
ROS: Reactive oxygen species
rhGH: Recombinant human GH
TRH: Thyrotropin-releasing hormone
TSH: Thyroid stimulating hormone
VIDD: Ventilator induced diaphragm dysfunction.

## References

[1] Loss SH Nunes DSL Franzosi OS Salazar GS Teixeira C Vieira SRR. Chronic critical illness: are we saving patients or creating victims? Rev Bras Ter Intensiva. (2017) 29:87–95. 10.5935/0103-507X.20170013 28444077 PMC5385990

[2] Van den Berghe G. Novel insights into the neuroendocrinology of critical illness. Eur J Endocrinol. (2000) 143:1–13. 10.1530/eje.0.1430001 10870025

[3] Nelson JE Cox CE Hope AA Carson SS. Chronic critical illness. Am J Respir Crit Care Med. (2010) 182:446–54. 10.1164/rccm.201002-0210CI 20448093 PMC2937238

[4] Van den Berghe G. Endocrine evaluation of patients with critical illness. Endocrinol Metab Clin North Am. (2003) 32:385–410. 10.1016/S0889-8529(03)00005-7 12800538

[5] Van den Berghe G. Novel insights in the HPA-axis during critical illness. Acta Clin Belg. (2014) 69:397–406. 10.1179/2295333714Y.0000000093 25409903

[6] Van den Berghe GH. Acute and prolonged critical illness are two distinct neuroendocrine paradigms. Verh K Acad Geneeskd Belg. (1998) 60:487–518; discussion −20. 10230322

[7] Vanhorebeek I Van den Berghe G. The neuroendocrine response to critical illness is a dynamic process. Crit Care Clin. (2006) 22:1–15, v. 10.1016/j.ccc.2005.09.004 16399016

[8] Van den Berghe G. On the neuroendocrinopathy of critical illness. perspectives for feeding and novel treatments. Am J Respir Crit Care Med. (2016) 194:1337–48. 10.1164/rccm.201607-1516CI 27611700

[9] Marik PE. Mechanisms and clinical consequences of critical illness associated adrenal insufficiency. Curr Opin Crit Care. (2007) 13:363–9. 10.1097/MCC.0b013e32818a6d74 17599004

[10] Boonen E Bornstein SR Van den Berghe G. New insights into the controversy of adrenal function during critical illness. Lancet Diabetes Endocrinol. (2015) 3:805–15. 10.1016/S2213-8587(15)00224-7 26071883

[11] Boelen A Kwakkel J Thijssen-Timmer DC Alkemade A Fliers E Wiersinga WM. Simultaneous changes in central and peripheral components of the hypothalamus-pituitary-thyroid axis in lipopolysaccharide-induced acute illness in mice. J Endocrinol. (2004) 182:315–23. 10.1677/joe.0.1820315 15283692

[12] Joseph-Bravo P Jaimes-Hoy L Charli JL. Regulation of TRH neurons and energy homeostasis-related signals under stress. J Endocrinol. (2015) 224:R139–59. 10.1530/JOE-14-0593 25563352

[13] Chatzitomaris A Hoermann R Midgley JE Hering S Urban A Dietrich B. Thyroid allostasis-adaptive responses of thyrotropic feedback control to conditions of strain, stress, and developmental programming. Front Endocrinol. (2017) 8:163. 10.3389/fendo.2017.00163 28775711 PMC5517413

[14] Harel G Shamoun DS Kane JP Magner JA Szabo M. Prolonged effects of tumor necrosis factor-alpha on anterior pituitary hormone release. Peptides. (1995) 16:641–5. 10.1016/0196-9781(95)00019-G 7479297

[15] Wassen FW Moerings EP Van Toor H De Vrey EA Hennemann G Everts ME. Effects of interleukin-1 beta on thyrotropin secretion and thyroid hormone uptake in cultured rat anterior pituitary cells. Endocrinology. (1996) 137:1591–8. 10.1210/endo.137.5.8612490 8612490

[16] Mancini A Di Segni C Raimondo S Olivieri G Silvestrini A Meucci E. Thyroid hormones, oxidative stress, and inflammation. Mediators Inflamm. (2016) 2016:6757154. 10.1155/2016/6757154 27051079 PMC4802023

[17] Van den Berghe G. Non-thyroidal illness in the ICU: a syndrome with different faces. Thyroid. (2014) 24:1456–65. 10.1089/thy.2014.0201 24845024 PMC4195234

[18] Peeters B Boonen E Langouche L Van den Berghe G. The HPA axis response to critical illness: New study results with diagnostic and therapeutic implications. Mol Cell Endocrinol. (2015) 408:235–40. 10.1016/j.mce.2014.11.012 25462585

[19] Téblick A Peeters B Langouche L Van den Berghe G. Adrenal function and dysfunction in critically ill patients. Nat Rev Endocrinol. (2019) 15:417–27. 10.1038/s41574-019-0185-7 30850749

[20] Van Aerde N Van Dyck L Vanhorebeek I Van den Berghe G. Endocrinopathy of the Critically Ill. In: Preiser J-C Herridge M Azoulay E editors. Post-Intensive Care Syndrome. Cham: Springer International Publishing (2020). p. 125–43. 10.1007/978-3-030-24250-3_9

[21] Rawal G Yadav S Kumar R. Post-intensive care syndrome: an overview. J Transl Int Med. (2017) 5:90–2. 10.1515/jtim-2016-0016 28721340 PMC5506407

[22] Komaroff AL. Advances in understanding the pathophysiology of chronic fatigue syndrome. JAMA. (2019) 322:499–500. 10.1001/jama.2019.8312 31276153

[23] Nacul L Authier J Scheibenbogen C Lorusso L Helland I Alegre Martin J. EUROPEAN ME NETWORK (EUROMENE) Expert Consensus on the Diagnosis, Service Provision and Care of People with ME/CFS in Europe [Preprint]. (2020). Available online at: https://www.preprints.org/manuscript/202009.0688/v2 (accessed March 27, 2021). 10.3390/medicina57050510 PMC8161074 34069603

[24] Chu L Valencia IJ Garvert DW Montoya JG. Onset patterns and course of myalgic encephalomyelitis/chronic fatigue syndrome. Front Pediatr. (2019) 7:12. 10.3389/fped.2019.00012 30805319 PMC6370741

[25] Institute of Medicine. Beyond Myalgic Encephalomyelitis/Chronic Fatigue Syndrome: Redefining an Illness. Washington, DC: The National Academies Press (2015). 25695122

[26] Open Medicine Foundation. 2020 Symptoms of ME/CFS. Available online at: https://www.omf.ngo/symptoms-mecfs (accessed March 27, 2021).

[27] Centers for Disease Control and Prevention. Clinical Care of Patients with ME/CFS - Severely Affected Patients. (2019). Available online at: https://www.cdc.gov/me-cfs/healthcare-providers/clinical-care-patients-mecfs/severely-affected-patients.html (accessed March 27, 2021).

[28] Nacul L O'Boyle S Palla L Nacul FE Mudie K Kingdon CC. How Myalgic Encephalomyelitis/Chronic Fatigue Syndrome (ME/CFS) progresses: the natural history of ME/CFS. Front Neurol. (2020) 11:826. 10.3389/fneur.2020.00826 32849252 PMC7431524

[29] Toogood PL Clauw DJ Phadke S Hoffman D. Myalgic encephalomyelitis/chronic fatigue syndrome (ME/CFS): where will the drugs come from? Pharmacol Res. (2021) 165:105465. 10.1016/j.phrs.2021.105465 33529750

[30] Richman S Morris MC Broderick G Craddock TJA Klimas NG Fletcher MA. Pharmaceutical interventions in chronic fatigue syndrome: a literature-based commentary. Clin Therap. (2019) 41:798–805. 10.1016/j.clinthera.2019.02.011 30871727 PMC6543846

[31] Kim DY Lee JS Park SY Kim SJ Son CG. Systematic review of randomized controlled trials for chronic fatigue syndrome/myalgic encephalomyelitis (CFS/ME). J Transl Med. (2020) 18:7. 10.1186/s12967-019-02196-9 31906979 PMC6943902

[32] Castro-Marrero J Sáez-Francàs N Santillo D Alegre J. Treatment and management of chronic fatigue syndrome/myalgic encephalomyelitis: all roads lead to Rome. Br J Pharmacol. (2017) 174:345–69. 10.1111/bph.13702 28052319 PMC5301046

[33] Rowe PC Underhill RA Friedman KJ Gurwitt A Medow MS Schwartz MS. Myalgic Encephalomyelitis/Chronic Fatigue syndrome diagnosis and management in young people: a primer. Front Pediatrics. (2017) 5:121. 10.3389/fped.2017.00121 28674681 PMC5474682

[34] Stanculescu D Larsson L Bergquist J. Hypothesis: mechanisms that prevent recovery in prolonged ICU patients also underlie Myalgic Encephalomyelitis/Chronic Fatigue Syndrome (ME/CFS). Front Med. (2021) 8:41. 10.3389/fmed.2021.628029 33585528 PMC7876311

[35] Meeus M Ickmans K Struyf F Kos D Lambrecht L Willekens B. What is in a name? Comparing diagnostic criteria for chronic fatigue syndrome with or without fibromyalgia. Clin Rheumatol. (2016) 35:191–203. 10.1007/s10067-014-2793-x 25308475

[36] Teodoro T Edwards MJ Isaacs JD. A unifying theory for cognitive abnormalities in functional neurological disorders, fibromyalgia and chronic fatigue syndrome: systematic review. J Neurol Neurosurg Psychiatry. (2018) 89:1308–19. 10.1136/jnnp-2017-317823 29735513 PMC6288708

[37] Natelson BH. Myalgic Encephalomyelitis/Chronic Fatigue Syndrome and fibromyalgia: definitions, similarities, and differences. Clin Ther. (2019) 41:612–8. 10.1016/j.clinthera.2018.12.016 30795933 PMC6589349

[38] Dennis A Wamil M Kapur S Alberts J Badley AD Decker GA. Multi-organ impairment in low-risk individuals with long COVID. medRxiv. (2020) :2020.10.14.20212555. 10.1101/2020.10.14.20212555

[39] Somasundaram NP Ranathunga I Ratnasamy V Wijewickrama PSA Dissanayake HA Yogendranathan N. The impact of SARS-Cov-2 virus infection on the endocrine system. J Endocrine Soc. (2020) 4:1–22. 10.1210/jendso/bvaa082 32728654 PMC7337839

[40] Greenhalgh T Knight M A'Court C Buxton M Husain L. Management of post-acute covid-19 in primary care. BMJ. (2020) 370:m3026. 10.1136/bmj.m3026 32784198

[41] Dani M Dirksen A Taraborrelli P Torocastro M Panagopoulos D Sutton R. Autonomic dysfunction in 'long COVID': rationale, physiology and management strategies. Clin Med. (2020) 21:e63–7. 10.7861/clinmed.2020-0896 33243837 PMC7850225

[42] Huang C Huang L Wang Y Li X Ren L Gu X. 6-month consequences of COVID-19 in patients discharged from hospital: a cohort study. Lancet. (2021) 397:220–32. 10.1016/S0140-6736(20)32656-8 33428867 PMC7833295

[43] Townsend L Dyer AH Jones K Dunne J Mooney A Gaffney F. Persistent fatigue following SARS-CoV-2 infection is common and independent of severity of initial infection. PLOS ONE. (2020) 15:e0240784. 10.1371/journal.pone.0240784 33166287 PMC7652254

[44] Komaroff AL Bateman L. Will COVID-19 lead to Myalgic Encephalomyelitis/Chronic Fatigue syndrome? Front Med. (2021) 7:1132. 10.3389/fmed.2020.606824 33537329 PMC7848220

[45] Wildwing T Holt N. The neurological symptoms of COVID-19: a systematic overview of systematic reviews, comparison with other neurological conditions and implications for healthcare services. Therap Adv Chronic Dis. (2021) 12:2040622320976979. 10.1177/2040622320976979 33796241 PMC7970685

[46] Comella PH Gonzalez-Kozlova E Kosoy R Charney AW Peradejordi IF Chandrasekar S. A molecular network approach reveals shared cellular and molecular signatures between chronic fatigue syndrome and other fatiguing illnesses. medRxiv. (2021) 10.1101/2021.01.29.21250755 33564792 PMC7872387

[47] Gheorghită V Barbu AE Gheorghiu ML Căruntu FA. Endocrine dysfunction in sepsis: a beneficial or deleterious host response? Germs. (2015) 5:17–25. 10.11599/germs.2015.1067 25763364 PMC4350863

[48] Boonen E Van den Berghe G. Endocrine responses to critical illness: novel insights and therapeutic implications. J Clin Endocrinol Metab. (2014) 99:1569–82. 10.1210/jc.2013-4115 24517153

[49] Poteliakhoff A. Adrenocortical activity and some clinical findings in acute and chronic fatigue. J Psychosomatic Res. (1981) 25:91–5. 10.1016/0022-3999(81)90095-7 6974238

[50] Demitrack MA Dale JK Straus SE Laue L Listwak SJ Kruesi MJ. Evidence for impaired activation of the hypothalamic-pituitary-adrenal axis in patients with chronic fatigue syndrome. J Clin Endocrinol Metab. (1991) 73:1224–34. 10.1210/jcem-73-6-1224 1659582

[51] Scott LV Medbak S Dinan TG. Blunted adrenocorticotropin and cortisol responses to corticotropin-releasing hormone stimulation in chronic fatigue syndrome. Acta Psychiatr Scand. (1998) 97:450–7. 10.1111/j.1600-0447.1998.tb10030.x 9669518

[52] Scott LV Medbak S Dinan TG. Desmopressin augments pituitary-adrenal responsivity to corticotropin-releasing hormone in subjects with chronic fatigue syndrome and in healthy volunteers. Biol Psychiatry. (1999) 45:1447–54. 10.1016/S0006-3223(98)00232-7 10356627

[53] Crofford LJ. The hypothalamic-pituitary-adrenal stress axis in fibromyalgia and chronic fatigue syndrome. Z Rheumatol. (1998) 57(Suppl. 2):67–71. 10.1007/s003930050239 10025087

[54] De Becker P De Meirleir K Joos E Campine I Van Steenberge E Smitz J. Dehydroepiandrosterone (DHEA) response to i.v. ACTH in patients with chronic fatigue syndrome. Horm Metab Res. (1999) 31:18–21. 10.1055/s-2007-978690 10077344

[55] Altemus M Dale JK Michelson D Demitrack MA Gold PW Straus SE. Abnormalities in response to vasopressin infusion in chronic fatigue syndrome. Psychoneuroendocrinology. (2001) 26:175–88. 10.1016/S0306-4530(00)00044-5 11087963

[56] Cleare AJ Miell J Heap E Sookdeo S Young L Malhi GS. Hypothalamo-pituitary-adrenal axis dysfunction in chronic fatigue syndrome, and the effects of low-dose hydrocortisone therapy. J Clin Endocrinol Metab. (2001) 86:3545–54. 10.1210/jcem.86.8.7735 11502777

[57] Cleare AJ Blair D Chambers S Wessely S. Urinary free cortisol in chronic fatigue syndrome. Am J Psychiatry. (2001) 158:641–3. 10.1176/appi.ajp.158.4.641 11282703

[58] Gaab J Huster D Peisen R Engert V Heitz V Schad T. Hypothalamic-pituitary-adrenal axis reactivity in chronic fatigue syndrome and health under psychological, physiological, and pharmacological stimulation. Psychosom Med. (2002) 64:951–62. 10.1097/00006842-200211000-00012 12461200

[59] Jerjes WK Cleare AJ Wessely S Wood PJ Taylor NF. Diurnal patterns of salivary cortisol and cortisone output in chronic fatigue syndrome. J Affect Disord. (2005) 87:299–304. 10.1016/j.jad.2005.03.013 15922454

[60] Segal TY Hindmarsh PC Viner RM. Disturbed adrenal function in adolescents with chronic fatigue syndrome. J Pediatr Endocrinol Metab. (2005) 18:295–301. 10.1515/JPEM.2005.18.3.295 15813608

[61] Van Den Eede F Moorkens G Van Houdenhove B Cosyns P Claes SJ. Hypothalamic-pituitary-adrenal axis function in chronic fatigue syndrome. Neuropsychobiology. (2007) 55:112–20. 10.1159/000104468 17596739

[62] Van Den Eede F Moorkens G Hulstijn W Van Houdenhove B Cosyns P Sabbe BG. Combined dexamethasone/corticotropin-releasing factor test in chronic fatigue syndrome. Psychol Med. (2008) 38:963–73. 10.1017/S0033291707001444 17803834

[63] Papadopoulos AS Cleare AJ. Hypothalamic-pituitary-adrenal axis dysfunction in chronic fatigue syndrome. Nat Rev Endocrinol. (2011) 8:22–32. 10.1038/nrendo.2011.153 21946893

[64] Craddock TJ Fritsch P Rice MA Jr. del Rosario RM Miller DB. A role for homeostatic drive in the perpetuation of complex chronic illness: Gulf War Illness and chronic fatigue syndrome. PLoS ONE. (2014) 9:e84839. 10.1371/journal.pone.0084839 24416298 PMC3885655

[65] Straub RH Schölmerich J Zietz B. Replacement therapy with DHEA plus corticosteroids in patients with chronic inflammatory diseases–substitutes of adrenal and sex hormones. Z Rheumatol. (2000) 59(Suppl. 2):Ii/108–18. 10.1007/PL00022854 11155790

[66] Kuratsune H Yamaguti K Sawada M Kodate S Machii T Kanakura Y. Dehydroepiandrosterone sulfate deficiency in chronic fatigue syndrome. Int J Mol Med. (1998) 1:143–6. 10.3892/ijmm.1.1.143 9852212

[67] Holtorf K. Diagnosis and treatment of hypothalamic-pituitary-adrenal (HPA) axis dysfunction in patients with Chronic Fatigue syndrome (CFS) and Fibromyalgia (FM). J Chronic Fatigue Syndrome. (2008) 14:59–88. 10.1300/J092v14n03_06

[68] Tomas C Newton J Watson S. A review of hypothalamic-pituitary-adrenal axis function in chronic fatigue syndrome. ISRN Neurosci. (2013) 2013:784520. 10.1155/2013/784520 24959566 PMC4045534

[69] Cleare AJ Heap E Malhi GS Wessely S O'Keane V Miell J. Low-dose hydrocortisone in chronic fatigue syndrome: a randomised crossover trial. Lancet. (1999) 353:455–8. 10.1016/S0140-6736(98)04074-4 9989716

[70] McKenzie R O'Fallon A Dale J Demitrack M Sharma G Deloria M. Low-dose hydrocortisone for treatment of chronic fatigue syndrome: a randomized controlled trial. JAMA. (1998) 280:1061–6. 10.1001/jama.280.12.1061 9757853

[71] Baschetti R. Low-dose hydrocortisone for chronic fatigue syndrome. JAMA. (1999) 281:1887–9. 10.1001/jama.281.20.1887 10349884

[72] Teitelbaum JE Bird B Weiss A Gould L. Low-dose hydrocortisone for chronic fatigue syndrome. Jama. (1999) 281(20):1887–8; author reply 8–9. 10349885

[73] Myhill S. Diagnosis and Treatment of Chronic Fatigue Syndrome and Myalgic Encephalitis, 2nd ed.: It's Mitochondria, Not Hypochondria. White River Junction, VT: Chelsea Green Publishing (2018) 432. p.

[74] Teitelbaum J. From Fatigued to Fantastic!: A Clinically Proven Program to Regain Vibrant Health and Overcome Chronic Fatigue and Fibromyalgia. New York, NY: Avery (2007). 424 p.

[75] Durrant-Peatfield B. Your Thyroid and how to Keep it Healthy. London: Hammersmith Press Limited (2006) 264. p.

[76] Skinner G. Diagnosis and Management of Hypothyroidism. West Midlands, UK: Louise Lorne. (2003) 201. p.

[77] Hertoghe T. Atlas of Endocrinology for Hormone Therapy. Luxembourg: International Medical Books. (2010).

[78] Honeyman-Lowe G Lowe JC. Your Guide to Metabolic Health. Boulder, CO: McDowell Health-Science Books, LLC (2003) 384. p.

[79] Bou-Holaigah I Rowe PC Kan J Calkins H. The relationship between neurally mediated hypotension and the chronic fatigue syndrome. JAMA. (1995) 274:961–7. 10.1001/jama.274.12.961 7674527

[80] Baschetti R. Chronic fatigue syndrome and neurally mediated hypotension. JAMA. (1996) 275:359–. 10.1001/jama.1996.03530290029022 8569008

[81] Baschetti R. Investigations of hydrocortisone and fludrocortisone in the treatment of chronic fatigue syndrome. J Clin Endocrinol Metab. (1999) 84:2263–4. 10.1210/jcem.84.6.5809-10 10372750

[82] Peterson PK Pheley A Schroeppel J Schenck C Marshall P Kind A. A preliminary placebo-controlled crossover trial of fludrocortisone for chronic fatigue syndrome. Arch Intern Med. (1998) 158:908–14. 10.1001/archinte.158.8.908 9570178

[83] Rowe PC Calkins H DeBusk K McKenzie R Anand R Sharma G. Fludrocortisone acetate to treat neurally mediated hypotension in chronic fatigue syndrome: a randomized controlled trial. JAMA. (2001) 285:52–9. 10.1001/jama.285.1.52 11150109

[84] Blockmans D Persoons P Van Houdenhove B Lejeune M Bobbaers H. Combination therapy with hydrocortisone and fludrocortisone does not improve symptoms in chronic fatigue syndrome: a randomized, placebo-controlled, double-blind, crossover study. Am J Med. (2003) 114:736–41. 10.1016/S0002-9343(03)00182-7 12829200

[85] Himmel PB Seligman TM. A pilot study employing Dehydroepiandrosterone (DHEA) in the treatment of chronic fatigue syndrome. J Clin Rheumatol. (1999) 5:56–9. 10.1097/00124743-199904000-00004 19078357

[86] Cabanas H Muraki K Balinas C Eaton-Fitch N Staines D Marshall-Gradisnik S. Validation of impaired transient receptor potential Melastatin 3 ion channel activity in natural killer cells from Chronic Fatigue Syndrome/ Myalgic Encephalomyelitis patients. Mol Med. (2019) 25:14. 10.1186/s10020-019-0083-4 31014226 PMC6480905

[87] Elijah IE Branski LK Finnerty CC Herndon DN. The GH/IGF-1 system in critical illness. Best Pract Res Clin Endocrinol Metab. (2011) 25:759–67. 10.1016/j.beem.2011.06.002 21925076 PMC3788574

[88] Weekers F Van den Berghe G. Endocrine modifications and interventions during critical illness. Proc Nutr Soc. (2004) 63:443–50. 10.1079/PNS2004373 15373956

[89] Takala J Ruokonen E Webster NR Nielsen MS Zandstra DF Vundelinckx G. Increased mortality associated with growth hormone treatment in critically ill adults. N Engl J Med. (1999) 341:785–92. 10.1056/NEJM199909093411102 10477776

[90] Teng Chung T Hinds CJ. Treatment with GH and IGF-1 in critical illness. Crit Care Clin. (2006) 22:29–40, vi. 10.1016/j.ccc.2005.09.003 16399018

[91] Hammarqvist F Wennström I Wernerman J. Effects of growth hormone and insulin-like growth factor-1 on postoperative muscle and substrate metabolism. J Nutr Metab. (2010) 2010:647929. 10.1155/2010/647929 20798757 PMC2925091

[92] Berwaerts J Moorkens G Abs R. Secretion of growth hormone in patients with chronic fatigue syndrome. Growth Horm IGF Res. (1998) 8(Suppl. B):127–9. 10.1016/S1096-6374(98)80036-1 10990147

[93] Moorkens G Berwaerts J Wynants H Abs R. Characterization of pituitary function with emphasis on GH secretion in the chronic fatigue syndrome. Clin Endocrinol. (2000) 53:99–106. 10.1046/j.1365-2265.2000.01049.x 10931086

[94] Moorkens G Wynants H Abs R. Effect of growth hormone treatment in patients with chronic fatigue syndrome: a preliminary study. Growth Horm IGF Res. (1998) 8(Suppl. B):131–3. 10.1016/S1096-6374(98)80037-3 10990148

[95] Bennett RM Clark SR Campbell SM Burckhardt CS. Low levels of somatomedin C in patients with the fibromyalgia syndrome. a possible link between sleep and muscle pain. Arthritis Rheum. (1992) 35:1113–6. 10.1002/art.1780351002 1418002

[96] Bennett RM Cook DM Clark SR Burckhardt CS Campbell SM. Hypothalamic-pituitary-insulin-like growth factor-I axis dysfunction in patients with fibromyalgia. J Rheumatol. (1997) 24:1384–9. 9228141

[97] Paiva ES Deodhar A Jones KD Bennett R. Impaired growth hormone secretion in fibromyalgia patients: evidence for augmented hypothalamic somatostatin tone. Arthritis Rheum. (2002) 46:1344–50. 10.1002/art.10209 12115242

[98] Gupta A Silman AJ. Psychological stress and fibromyalgia: a review of the evidence suggesting a neuroendocrine link. Arthritis Res Ther. (2004) 6:98–106. 10.1186/ar1176 15142258 PMC416451

[99] Cuatrecasas G Gonzalez MJ Alegre C Sesmilo G Fernandez-Sola J Casanueva FF. High prevalence of growth hormone deficiency in severe fibromyalgia syndromes. J Clin Endocrinol Metab. (2010) 95:4331–7. 10.1210/jc.2010-0061 20631018

[100] Rigamonti AE Grugni G Arreghini M Capodaglio P De Col A Agosti F. GH responsiveness to combined gh-releasing hormone and arginine administration in obese patients with fibromyalgia syndrome. Int J Endocrinol. (2017) 2017:3106041. 10.1155/2017/3106041 28744309 PMC5506478

[101] Riedel W Schlapp U Leck S Netter P Neeck G. Blunted ACTH and cortisol responses to systemic injection of corticotropin-releasing hormone (CRH) in fibromyalgia: role of somatostatin and CRH-binding protein. Ann N Y Acad Sci. (2002) 966:483–90. 10.1111/j.1749-6632.2002.tb04251.x 12114308

[102] Bennett RM Clark SC Walczyk J. A randomized, double-blind, placebo-controlled study of growth hormone in the treatment of fibromyalgia. Am J Med. (1998) 104:227–31. 10.1016/S0002-9343(97)00351-3 9552084

[103] Cuatrecasas G Riudavets C Güell MA Nadal A. Growth hormone as concomitant treatment in severe fibromyalgia associated with low IGF-1 serum levels. a pilot study. BMC Musculoskeletal Disorders. (2007) 8:119. 10.1186/1471-2474-8-119 18053120 PMC2212629

[104] Cuatrecasas G Alegre C Casanueva FF. GH/IGF1 axis disturbances in the fibromyalgia syndrome: is there a rationale for GH treatment? Pituitary. (2014) 17:277–83. 10.1007/s11102-013-0486-0 23568565

[105] Cuatrecasas G Alegre C Fernandez-Solà J Gonzalez MJ Garcia-Fructuoso F Poca-Dias V. Growth hormone treatment for sustained pain reduction and improvement in quality of life in severe fibromyalgia. Pain. (2012) 153:1382–9. 10.1016/j.pain.2012.02.012 22465047

[106] Carter JN Eastman CJ Corcoran JM Lazarus L. Effects of triiodothyronine administration in patients with chronic renal failure. Aust N Z J Med. (1977) 7:612–6. 10.1111/j.1445-5994.1977.tb02317.x 274939

[107] Brent GA Hershman JM. Thyroxine therapy in patients with severe nonthyroidal illnesses and low serum thyroxine concentration. J Clin Endocrinol Metab. (1986) 63:1–8. 10.1210/jcem-63-1-1 3011834

[108] De Groot LJ. Dangerous dogmas in medicine: the nonthyroidal illness syndrome. J Clin Endocrinol Metab. (1999) 84:151–64. 10.1210/jcem.84.1.5364 9920076

[109] Langouche L Jacobs A Van den Berghe G. Nonthyroidal illness syndrome across the ages. J Endocrine Soc. (2019) 3:2313–25. 10.1210/js.2019-00325 31745528 PMC6853682

[110] Peeters RP van der Geyten S Wouters PJ Darras VM van Toor H Kaptein E. Tissue thyroid hormone levels in critical illness. J Clin Endocrinol Metab. (2005) 90:6498–507. 10.1210/jc.2005-1013 16174716

[111] Davis PJ. Cytokines and growth factors and thyroid hormone. Curr Opin Endocrinol Diabetes Obes. (2008) 15:428. 10.1097/MED.0b013e32830eba0e 18769214

[112] Kaptein EM Sanchez A Beale E Chan LS. Clinical review: thyroid hormone therapy for postoperative nonthyroidal illnesses: a systematic review and synthesis. J Clin Endocrinol Metab. (2010) 95:4526–34. 10.1210/jc.2010-1052 20668034

[113] De Groot LJ. The non-thyroidal illness syndrome [Updated 2015 Feb 1]. In: Feingold KR Anawalt B Boyce A Chrousos G de Herder WW Dungan K. editors. Endotext [Internet]. South Dartmouth, MA: MDText.com, Inc. (2000).

[114] Moura Neto A Zantut-Wittmann DE. Abnormalities of thyroid hormone metabolism during systemic illness: the low T3 syndrome in different clinical settings. Int J Endocrinol. (2016) 2016:2157583. 10.1155/2016/2157583 27803712 PMC5075641

[115] Breitzig MT Alleyn MD Lockey RF Kolliputi N. Thyroid hormone: a resurgent treatment for an emergent concern. Am J Physiol Lung Cell Mol Physiol. (2018) 315:L945–L50. 10.1152/ajplung.00336.2018 30260285 PMC6337010

[116] Farwell AP. Thyroid hormone therapy is not indicated in the majority of patients with the sick euthyroid syndrome. Endocr Pract. (2008) 14:1180–7. 10.4158/EP.14.9.1180 19158057

[117] Fliers E Bianco AC Langouche L Boelen A. Thyroid function in critically ill patients. Lancet Diabetes Endocrinol. (2015) 3:816–25. 10.1016/S2213-8587(15)00225-9 26071885 PMC4979220

[118] Pappa TA Vagenakis AG Alevizaki M. The nonthyroidal illness syndrome in the non-critically ill patient. Europ J Clin Invest. (2011) 41:212–20. 10.1111/j.1365-2362.2010.02395.x 20964678

[119] Warner MH Beckett GJ. Mechanisms behind the non-thyroidal illness syndrome: an update. J Endocrinol. (2010) 205:1–13. 10.1677/JOE-09-0412 20016054

[120] Wajner SM Maia AL. New insights toward the acute non-thyroidal illness syndrome. Front Endocrinol. (2012) 3:8. 10.3389/fendo.2012.00008 22654851 PMC3356062

[121] Biondi B Wartofsky L. Treatment with thyroid hormone. Endocr Rev. (2014) 35:433–512. 10.1210/er.2013-1083 24433025

[122] Somppi TL. Non-thyroidal illness syndrome in patients exposed to indoor air dampness microbiota treated successfully with triiodothyronine. Front Immunol. (2017) 8:919. 10.3389/fimmu.2017.00919 28824644 PMC5545575

[123] Adler SM Wartofsky L. The nonthyroidal illness syndrome. Endocrinol Metab Clin North Am. (2007) 36:657–72, vi. 10.1016/j.ecl.2007.04.007 17673123

[124] Bianco AC. Minireview: cracking the metabolic code for thyroid hormone signaling. Endocrinology. (2011) 152:3306–11. 10.1210/en.2011-1104 21712363 PMC3159779

[125] Peeters R Visser T. Metabolism of thyroid hormone. [Updated 2017 Jan 1]. In: Feingold KR Anawalt B Boyce A Chrousos G de Herder WW Dungan K. editors. *Endotext [Internet].* South Dartmouth, MA: MDText.com, Inc. (2000).

[126] Russell W Harrison RF Smith N Darzy K Shalet S Weetman AP. Free triiodothyronine has a distinct circadian rhythm that is delayed but parallels thyrotropin levels. J Clin Endocrinol Metab. (2008) 93:2300–6. 10.1210/jc.2007-2674 18364382

[127] Robinson P. Recovering With T3: My Journey From Hypothyroidism to Good Health Using the T3 Thyroid Hormone. Nailsea: Elephant in the Room Books (2018) 288. p.

[128] Kansagra SM McCudden CR Willis MS. The challenges and complexities of thyroid hormone replacement. Lab Med. (2010) 41:338–48. 10.1309/LMB39TH2FZGNDGIM

[129] Gereben B Zavacki AM Ribich S Kim BW Huang SA Simonides WS. Cellular and molecular basis of deiodinase-regulated thyroid hormone signaling. Endocr Rev. (2008) 29:898–938. 10.1210/er.2008-0019 18815314 PMC2647704

[130] Debaveye Y Ellger B Mebis L Visser TJ Darras VM Van den Berghe G. Effects of substitution and high-dose thyroid hormone therapy on deiodination, sulfoconjugation, and tissue thyroid hormone levels in prolonged critically ill rabbits. Endocrinology. (2008) 149:4218–28. 10.1210/en.2007-1566 18450965 PMC2488214

[131] Lado-Abeal J Romero A Castro-Piedras I Rodriguez-Perez A Alvarez-Escudero J. Thyroid hormone receptors are down-regulated in skeletal muscle of patients with non-thyroidal illness syndrome secondary to non-septic shock. Eur J Endocrinol. (2010) 163:765–73. 10.1530/EJE-10-0376 20736347

[132] Dietrich JW Landgrafe-Mende G Wiora E Chatzitomaris A Klein HH Midgley JE. Calculated parameters of thyroid homeostasis: emerging tools for differential diagnosis and clinical research. Front Endocrinol. (2016) 7:57. 10.3389/fendo.2016.00057 27375554 PMC4899439

[133] Fuite J Vernon SD Broderick G. Neuroendocrine and immune network re-modeling in chronic fatigue syndrome: an exploratory analysis. Genomics. (2008) 92:393–9. 10.1016/j.ygeno.2008.08.008 18775774

[134] Ruiz-Núñez B Tarasse R Vogelaar EF Janneke Dijck-Brouwer DA Muskiet FAJ. Higher prevalence of “Low T3 Syndrome” in patients with chronic fatigue syndrome: a case-control study. Front Endocrinol. (2018) 9:97. 10.3389/fendo.2018.00097 29615976 PMC5869352

[135] Lowe JC Garrison RL Reichman AJ Yellin J Thompson M Kaufman D. Effectiveness and safety of T3 (Triiodothyronine) therapy for euthyroid fibromyalgia. Clin Bull Myofascial Therap. (1996) 2:31–57. 10.1300/J425v02n02_04

[136] Lowe JC Reichman AJ Yellin J. The process of change during T3 treatment for euthyroid fibromyalgia. Clin Bull Myofascial Therap. (1996) 2:91–124. 10.1300/J425v02n02_07

[137] Lowe JC. Results of an open trial of T3 therapy with 77 euthyroid female fibromyalgia patients. Clin Bull Myofascial Therap. (1996) 2:35–7. 10.1300/J425v02n01_04

[138] Lowe JC Garrison RL Reichman A Yellin J. Triiodothyronine (T3) treatment of euthyroid fibromyalgia. Clin Bull Myofascial Therap. (1997) 2:71–88. 10.1300/J425v02n04_05

[139] Lowe JC Yellin JG. The Metabolic Treatment of Fibromyalgia. Boulder, CO: McDowell Publishing Company (2000) 1260. p.

[140] Teitelbaum JE Bird B Greenfield RM Weiss A Muenz L Gould L. Effective treatment of chronic fatigue syndrome and fibromyalgia-a randomized, double-blind, placebo-controlled, intent-to-treat study. J Chronic Fatigue Syndrome. (2000) 8:3–15. 10.1300/J092v08n02_02

[141] Wharton GK. Unrecognized hypothyroidism. Can Med Assoc J. (1939) 40:371–6. 20321305 PMC537100

[142] Barnes B. Hypothyroidism: The Unsuspected Illness. New York, NY: HarperCollins. (1976).

[143] Wilson ED. Wilson's Syndrome: The Miracle of Feeling Well. Houston, TX: Cornerstone Publishing Company (1991) 346. p.

[144] Teitelbaum J Bird B. Effective treatment of severe chronic fatigue: a report of a series of 64 patients. J Musculoskeletal Pain. (1995) 3:91–110. 10.1300/J094v03n04_11

[145] Claeys B Hertoghe T. En finir avec l'hypothyroïdie: ce que votre médecin ne vous dit pas et que vous devez savoir. Vergèze: Thierry Souccar Editions (2015).

[146] Skinner GR Thomas R Taylor M Sellarajah M Bolt S Krett S. Thyroxine should be tried in clinically hypothyroid but biochemically euthyroid patients. BMJ. (1997) 314:1764. 10.1136/bmj.314.7096.1764 9202524 PMC2126906

[147] Holmes D. Tears Behind Closed Doors: Failure to Diagnose a Thyroid Condition. Wolverhampton: Normandi Publishing Limited. (2002) 270. p.

[148] Bowthorpe JA. Stop the Thyroid Madness: A Patient Revolution Against Decades of Inferior Thyroid Treatment. Fredericksburg, TX: Laughing Grape Pub. (2008) 293. p.

[149] Stanculescu D. Pure T3 Thyroid and Stories of Recovery from Chronic Fatigue Syndrome (ME/CFS) and Fibromyalgia: An Overview [Internet]. Health Rising (2019). Available online at: https://www.healthrising.org/blog/2019/03/07/thyroid-t3-chronic-fatigue-fibromyalgia-recovery-stories/ (accessed March 27, 2021).

[150] Sánchez-Franco F Fernández L Fernández G Cacicedo L. Thyroid hormone action on ACTH secretion. Horm Metab Res. (1989) 21:550–2. 10.1055/s-2007-1009285 2553572

[151] Lizcano F Rodríguez JS. Thyroid hormone therapy modulates hypothalamo-pituitary-adrenal axis. Endocr J. (2011) 58:137–42. 10.1507/endocrj.K10E-369 21263198

[152] Wikland B Löwhagen T Sandberg PO. Fine-needle aspiration cytology of the thyroid in chronic fatigue. Lancet. (2001) 357:956–7. 10.1016/S0140-6736(05)71654-8 11289370

[153] Sotzny F Blanco J Capelli E Castro-Marrero J Steiner S Murovska M. Myalgic Encephalomyelitis/Chronic Fatigue syndrome - evidence for an autoimmune disease. Autoimmun Rev. (2018) 17:601–9. 10.1016/j.autrev.2018.01.009 29635081

[154] Castro-Marrero J Faro M Aliste L Sáez-Francàs N Calvo N Martínez-Martínez A. Comorbidity in Chronic Fatigue Syndrome/Myalgic Encephalomyelitis: A nationwide population-based cohort study. Psychosomatics. (2017) 58:533–43. 10.1016/j.psym.2017.04.010 28596045

[155] Midgley JE Larisch R Dietrich JW Hoermann R. Variation in the biochemical response to l-thyroxine therapy and relationship with peripheral thyroid hormone conversion efficiency. Endocr Connect. (2015) 4:196–205. 10.1530/EC-150056 26335522 PMC4557078

[156] Midgley JEM Toft AD Larisch R Dietrich JW Hoermann R. Time for a reassessment of the treatment of hypothyroidism. BMC Endocrine Disord. (2019) 19:37. 10.1186/s12902-019-0365-4 30999905 PMC6471951

[157] Gereben B McAninch EA Ribeiro MO Bianco AC. Scope and limitations of iodothyronine deiodinases in hypothyroidism. Nat Rev Endocrinol. (2015) 11:642–52. 10.1038/nrendo.2015.155 26416219 PMC5003781

[158] Werneck de Castro JP Fonseca TL Ueta CB McAninch EA Abdalla S Wittmann G. Differences in hypothalamic type 2 deiodinase ubiquitination explain localized sensitivity to thyroxine. J Clin Invest. (2015) 125:769–81. 10.1172/JCI77588 25555216 PMC4319436

[159] McAninch EA Bianco AC. The history and future of treatment of hypothyroidism. Ann Intern Med. (2016) 164:50–6. 10.7326/M15-1799 26747302 PMC4980994

[160] Chaker L Bianco AC Jonklaas J Peeters RP. Hypothyroidism. Lancet. (2017) 390:1550–62. 10.1016/S0140-6736(17)30703-1 28336049 PMC6619426

[161] Smith T. Thyroid Hormone Journey: Metabolism [Internet]. Thyroid Patients Canada (2021). Available online at: https://thyroidpatients.ca/2021/01/31/journey-metabolism/ (accessed March 27, 2021).

[162] Hoermann R Midgley JEM Larisch R Dietrich JW. Individualised requirements for optimum treatment of hypothyroidism: complex needs, limited options. Drugs Context. (2019) 8:212597. 10.7573/dic.212597 31516533 PMC6726361

[163] Larisch R Midgley JEM Dietrich JW Hoermann R. Symptomatic relief is related to serum free triiodothyronine concentrations during follow-up in levothyroxine-treated patients with differentiated thyroid cancer. Exp Clin Endocrinol Diabetes. (2018) 126:546–52. 10.1055/s-0043-125064 29396968

[164] Carlé A Faber J Steffensen R Laurberg P Nygaard B. hypothyroid patients encoding combined MCT10 and DIO2 gene polymorphisms may prefer L-T3 + L-T4 combination treatment - data using a blind, randomized, clinical study. Europ Thyroid J. (2017) 6:143–51. 10.1159/000469709 28785541 PMC5527224

[165] Park E Jung J Araki O Tsunekawa K Park SY Kim J. Concurrent TSHR mutations and DIO2 T92A polymorphism result in abnormal thyroid hormone metabolism. Sci Rep. (2018) 8:10090. 10.1038/s41598-018-28480-0 29973617 PMC6031622

[166] Paragliola RM Corsello A Concolino P Ianni F Papi G Pontecorvi A. Iodothyronine deiodinases and reduced sensitivity to thyroid hormones. Front Biosci. (2020) 25:201–28. 10.2741/4803 31585886

[167] Giustina A Bussi AR Jacobello C Wehrenberg WB. Effects of recombinant human growth hormone (GH) on bone and intermediary metabolism in patients receiving chronic glucocorticoid treatment with suppressed endogenous GH response to GH-releasing hormone. J Clin Endocrinol Metab. (1995) 80:122–9. 10.1210/jcem.80.1.7829600 7829600

[168] Oehri M Ninnis R Girard J Frey FJ Keller U. Effects of growth hormone and IGF-I on glucocorticoid-induced protein catabolism in humans. Am J Physiol. (1996) 270:E552–8. 10.1152/ajpendo.1996.270.4.E552 8928758

[169] Baxter RC. Changes in the IGF-IGFBP axis in critical illness. Best Pract Res Clin Endocrinol Metab. (2001) 15:421–34. 10.1053/beem.2001.0161 11800515

[170] Riedel W Layka H Neeck G. Secretory pattern of GH, TSH, thyroid hormones, ACTH, cortisol, FSH, and LH in patients with fibromyalgia syndrome following systemic injection of the relevant hypothalamic-releasing hormones. Z Rheumatol. (1998) 57(Suppl. 2):81–7. 10.1007/s003930050242 10025090

[171] Komesaroff PA Esler MD Sudhir K. Estrogen supplementation attenuates glucocorticoid and catecholamine responses to mental stress in perimenopausal women. J Clin Endocrinol Metab. (1999) 84:606–10. 10.1210/jc.84.2.606 10022424

[172] Gräns H Nilsson M Dahlman-Wright K Evengård B. Reduced levels of oestrogen receptor beta mRNA in Swedish patients with chronic fatigue syndrome. J Clin Pathol. (2007) 60:195–8. 10.1136/jcp.2005.035956 16731592 PMC1860629

[173] Samuels MH. Effects of variations in physiological cortisol levels on thyrotropin secretion in subjects with adrenal insufficiency: a clinical research center study1. J Clin Endocrinol Metab. (2000) 85:1388–93. 10.1210/jcem.85.4.6540 10770171

[174] Peeters B Meersseman P Vander Perre S Wouters PJ Debaveye Y Langouche L. ACTH and cortisol responses to CRH in acute, subacute, and prolonged critical illness: a randomized, double-blind, placebo-controlled, crossover cohort study. Intensive Care Med. (2018) 44:2048–58. 10.1007/s00134-018-5427-y 30374692 PMC6280831

[175] Boonen E Langouche L Janssens T Meersseman P Vervenne H De Samblanx E. Impact of duration of critical illness on the adrenal glands of human intensive care patients. J Clin Endocrinol Metab. (2014) 99:4214–22. 10.1210/jc.2014-2429 25062464

[176] Younes AK Younes NK. Recovery of steroid induced adrenal insufficiency. Transl Pediatr. (2017) 6:269–73. 10.21037/tp.2017.10.01 29184808 PMC5682381

[177] Nicolas C Nicolaides ANP Maria Alexandra Maria Alexandra George P Chrousos. Glucocorticoid therapy and adrenal suppression. [Updated 2018 Oct 19]. In: Feingold KR Anawalt B Boyce A Chrousos G de Herder WW Dungan K. editors. Endotext [Internet]. South Dartmouth, MA: MDText.com, Inc. (2000).

[178] Kannan CR. The Adrenal Gland. New York, NY: Springer-Verlag New York Inc. (2011).

[179] Jehan Abdulla Torpy B. Chronic Fatigue Syndrome. [Updated 2017 Apr 20]. In: Feingold KR Anawalt B Boyce A Chrousos G de Herder WW Dungan K. editors. Endotext [Internet]. South Dartmouth, MA: MDText.com, Inc. (2000).

[180] Bearn J Allain T Coskeran P Munro N Butler J McGregor A. Neuroendocrine responses to d-fenfluramine and insulin-induced hypoglycemia in chronic fatigue syndrome. Biol Psychiatry. (1995) 37:245–52. 10.1016/0006-3223(94)00121-I 7711161

[181] Scott LV Burnett F Medbak S Dinan TG. Naloxone-mediated activation of the hypothalamic-pituitary-adrenal axis in chronic fatigue syndrome. Psychol Med. (1998) 28:285–93. 10.1017/S0033291797006260 9572086

[182] Scott LV Teh J Reznek R Martin A Sohaib A Dinan TG. Small adrenal glands in chronic fatigue syndrome: a preliminary computer tomography study. Psychoneuroendocrinology. (1999) 24:759–68. 10.1016/S0306-4530(99)00028-1 10451910

[183] Gupta S Aslakson E Gurbaxani BM Vernon SD. Inclusion of the glucocorticoid receptor in a hypothalamic pituitary adrenal axis model reveals bistability. Theor Biol Med Model. (2007) 4:8. 10.1186/1742-4682-4-8 17300722 PMC1804264

[184] Ben-Zvi A Vernon SD Broderick G. Model-based therapeutic correction of hypothalamic-pituitary-adrenal axis dysfunction. PLOS Comput Biol. (2009) 5:e1000273. 10.1371/journal.pcbi.1000273 19165314 PMC2613527

[185] Sedghamiz H Morris M Craddock TJA Whitley D Broderick G. High-fidelity discrete modeling of the HPA axis: a study of regulatory plasticity in biology. BMC Syst Biol. (2018) 12:76. 10.1186/s12918-018-0599-1 30016990 PMC6050677

[186] Craddock TJ Del Rosario RR Rice M Zysman JP Fletcher MA Klimas NG. Achieving remission in gulf war illness: a simulation-based approach to treatment design. PLoS ONE. (2015) 10:e0132774. 10.1371/journal.pone.0132774 26192591 PMC4508058

[187] Zarzer CA Puchinger MG Kohler G Kugler P. Differentiation between genomic and non-genomic feedback controls yields an HPA axis model featuring hypercortisolism as an irreversible bistable switch. Theor Biol Med Model. (2013) 10:65. 10.1186/1742-4682-10-65 24209391 PMC3879227

[188] Hosseinichimeh N Rahmandad H Wittenborn AK. Modeling the hypothalamus-pituitary-adrenal axis: A review and extension. Math Biosci. (2015) 268:52–65. 10.1016/j.mbs.2015.08.004 26277048 PMC4568136

[189] Morris MC Cooney KE Sedghamiz H Abreu M Collado F Balbin EG. Leveraging prior knowledge of endocrine immune regulation in the therapeutically relevant phenotyping of women with chronic fatigue syndrome. Clin Ther. (2019) 41:656–74 e4. 10.1016/j.clinthera.2019.03.002 30929860 PMC6478538

[190] Kim JH Choi MH. Embryonic development and adult regeneration of the adrenal gland. Endocrinol Metab. (2020) 35:765–73. 10.3803/EnM.2020.403 33397037 PMC7803617

[191] Cleare AJ O'Keane V Miell JP. Levels of DHEA and DHEAS and responses to CRH stimulation and hydrocortisone treatment in chronic fatigue syndrome. Psychoneuroendocrinology. (2004) 29:724–32. 10.1016/S0306-4530(03)00104-5 15110921

[192] Inder WJ Prickett TC Mulder RT. Normal opioid tone and hypothalamic-pituitary-adrenal axis function in chronic fatigue syndrome despite marked functional impairment. Clin Endocrinol. (2005) 62:343–8. 10.1111/j.1365-2265.2005.02220.x 15730417

[193] Griep EN Boersma JW de Kloet ER. Altered reactivity of the hypothalamic-pituitary-adrenal axis in the primary fibromyalgia syndrome. J Rheumatol. (1993) 20:469–74. 8386766

[194] Scott LV Medbak S Dinan TG. The low dose ACTH test in chronic fatigue syndrome and in health. Clin Endocrinol. (1998) 48:733–7. 10.1046/j.1365-2265.1998.00418.x 9713562

[195] Van den Berghe G de Zegher F Baxter RC Veldhuis JD Wouters P Schetz M. Neuroendocrinology of prolonged critical illness: effects of exogenous thyrotropin-releasing hormone and its combination with growth hormone secretagogues. J Clin Endocrinol Metab. (1998) 83:309–19. 10.1210/jc.83.2.309 9467533

[196] Van den Berghe G Wouters P Weekers F Mohan S Baxter RC Veldhuis JD. Reactivation of pituitary hormone release and metabolic improvement by infusion of growth hormone-releasing peptide and thyrotropin-releasing hormone in patients with protracted critical illness. J Clin Endocrinol Metab. (1999) 84:1311–23. 10.1210/jc.84.4.1311 10199772

[197] Fisher DA. Physiological variations in thyroid hormones: physiological and pathophysiological considerations. Clin Chem. (1996) 42:135–9. 10.1093/clinchem/42.1.135 8565215

[198] Mesotten D Van den Berghe G. Changes within the growth hormone/insulin-like growth factor I/IGF binding protein axis during critical illness. Endocrinol Metab Clin North Am. (2006) 35:793–805, ix–x. 10.1016/j.ecl.2006.09.010 17127147

[199] Cheung AM Tansey CM Tomlinson G Diaz-Granados N Matté A Barr A. Two-year outcomes, health care use, and costs of survivors of acute respiratory distress syndrome. Am J Respir Crit Care Med. (2006) 174:538–44. 10.1164/rccm.200505-693OC 16763220

[200] Herridge MS. Recovery and long-term outcome in acute respiratory distress syndrome. Crit Care Clin. (2011) 27:685–704. 10.1016/j.ccc.2011.04.003 21742223

[201] Herridge MS Cheung AM Tansey CM Matte-Martyn A Diaz-Granados N Al-Saidi F. One-year outcomes in survivors of the acute respiratory distress syndrome. N Engl J Med. (2003) 348:683–93. 10.1056/NEJMoa022450 12594312

[202] Herridge MS Tansey CM Matté A Tomlinson G Diaz-Granados N Cooper A. Functional disability 5 years after acute respiratory distress syndrome. N Engl J Med. (2011) 364:1293–304. 10.1056/NEJMoa1011802 21470008

[203] Cleare AJ Sookdeo SS Jones J O'Keane V Miell JP. Integrity of the growth hormone/insulin-like growth factor system is maintained in patients with chronic fatigue syndrome. J Clin Endocrinol Metab. (2000) 85:1433–9. 10.1210/jc.85.4.1433 10770178

[204] Jones KD Burckhardt CS Deodhar AA Perrin NA Hanson GC Bennett RM. A six-month randomized controlled trial of exercise and pyridostigmine in the treatment of fibromyalgia. Arthritis Rheum. (2008) 58:612–22. 10.1002/art.23203 18240245 PMC2542941

[205] Van den Berghe G Weekers F Baxter RC Wouters P Iranmanesh A Bouillon R. Five-day pulsatile gonadotropin-releasing hormone administration unveils combined hypothalamic-pituitary-gonadal defects underlying profound hypoandrogenism in men with prolonged critical illness. J Clin Endocrinol Metab. (2001) 86:3217–26. 10.1210/jc.86.7.3217 11443192

[206] Van den Berghe G Baxter RC Weekers F Wouters P Bowers CY Iranmanesh A. The combined administration of GH-releasing peptide-2 (GHRP-2), TRH and GnRH to men with prolonged critical illness evokes superior endocrine and metabolic effects compared to treatment with GHRP-2 alone. Clin Endocrinol (Oxf). (2002) 56:655–69. 10.1046/j.1365-2265.2002.01255.x 12030918

[207] Anstey M Desai S Torre L Wibrow B Seet J Osnain E. Anabolic steroid use for weight and strength gain in critically ill patients: a case series and review of the literature. Case Rep Crit Care. (2018) 2018:4545623. 10.1155/2018/4545623 29854477 PMC5964539

[208] Mackay A Tate WP. A compromised paraventricular nucleus within a dysfunctional hypothalamus: a novel neuroinflammatory paradigm for ME/CFS. Int J Immunopathol Pharmacol. (2018) 32:2058738418812342. 10.1177/2058738418812342

[209] Morris G Maes M. Mitochondrial dysfunctions in myalgic encephalomyelitis/chronic fatigue syndrome explained by activated immuno-inflammatory, oxidative and nitrosative stress pathways. Metab Brain Dis. (2014) 29:19–36. 10.1007/s11011-013-9435-x 24557875

[210] Armstrong CW McGregor NR Lewis DP Butt HL Gooley PR. Metabolic profiling reveals anomalous energy metabolism and oxidative stress pathways in chronic fatigue syndrome patients. Metabolomics. (2015) 11:1626–39. 10.1007/s11306-015-0816-5

[211] Pall M. The NO/ONOO-cycle mechanism as the cause of chronic fatigue syndrome/myalgia encephalomyelitis. In: Svoboda E Zelenjcik K editors. Chronic Fatigue Syndrome: Symptoms, Causes and Prevention. Hauppauge, NY: Nova Publishers (2009) p. Chapter 2.

[212] Shungu DC Weiduschat N Murrough JW Mao X Pillemer S Dyke JP. Increased ventricular lactate in chronic fatigue syndrome. III. Relationships to cortical glutathione and clinical symptoms implicate oxidative stress in disorder pathophysiology. NMR Biomed. (2012) 25:1073–87. 10.1002/nbm.2772 22281935 PMC3896084

[213] Morris G Maes M. Oxidative and nitrosative stress and immune-inflammatory pathways in patients with Myalgic Encephalomyelitis (ME)/Chronic Fatigue Syndrome (CFS). Curr Neuropharmacol. (2014) 12:168–85. 10.2174/1570159X11666131120224653 24669210 PMC3964747

[214] Montoya JG Holmes TH Anderson JN Maecker HT Rosenberg-Hasson Y Valencia IJ. Cytokine signature associated with disease severity in chronic fatigue syndrome patients. Proc Natl Acad Sci USA. (2017) 114:E7150–E8. 10.1073/pnas.1710519114 28760971 PMC5576836

[215] Hornig M Montoya JG Klimas NG Levine S Felsenstein D Bateman L. Distinct plasma immune signatures in ME/CFS are present early in the course of illness. Sci Adv. (2015) 1:e1400121. 10.1126/sciadv.1400121 26079000 PMC4465185

[216] Moutschen M Triffaux JM Demonty J Legros JJ Lefèbvre PJ. Pathogenic tracks in fatigue syndromes. Acta Clin Belg. (1994) 49:274–89. 10.1080/17843286.1994.11718404 7871934

[217] Bianco AC Kim BW. Deiodinases: implications of the local control of thyroid hormone action. J Clin Invest. (2006) 116:2571–9. 10.1172/JCI29812 17016550 PMC1578599

[218] Neeck G Riedel W. Thyroid function in patients with fibromyalgia syndrome. The Journal of rheumatology. (1992) 19:1120–2.- 1512769

[219] Neeck G Crofford LJ. Neuroendocrine peturbations in fibromyalgia and chronic fatigue syndrome. Rheum Dis Clin North America. (2000) 26:989–1002. 10.1016/S0889-857X(05)70180-0 11084955

[220] Lowe JC. Thyroid status of 38 fibromyalgia patients. Clin Bull Myofascial Therap. (1996) 2:47–64. 10.1300/J425v02n01_07

[221] Lowe JC Reichman AJ Honeyman GS Yellin J. Thyroid status of fibromyalgia patients. Clin Bull Myofascial Therap. (1998) 3:69–70. 10.1300/J425v03n01_08

[222] Garrison RL Breeding PC. A metabolic basis for fibromyalgia and its related disorders: the possible role of resistance to thyroid hormone. Med Hypotheses. (2003) 61:182–9. 10.1016/S0306-9877(02)00294-3 12888300

[223] Lowe JC Yellin J. Inadequate thyroid hormone regulation as the main mechanism of fibromyalgia: a review of the evidence. Thyroid Sci. (2008) 3:R1–14. Available online at: http://citeseerx.ist.psu.edu/viewdoc/summary?doi=10.1.1.627.459

[224] Holtorf K. Thyroid hormone transport into cellular tissue. J Restorative Med. (2014) 3:53–68. 10.14200/jrm.2014.3.0104

[225] Holtorf K. Peripheral thyroid hormone conversion and its impact on TSH and metabolic activity. J Restorative Med. (2014) 3:30–52. 10.14200/jrm.2014.3.0103

[226] Cioffi F Senese R Petito G Lasala P de Lange P Silvestri E. Both 3,3′,5-triiodothyronine and 3,5-diodo-L-thyronine are able to repair Mitochondrial DNA damage but by different mechanisms. Front Endocrinol. (2019) 10:216. 10.3389/fendo.2019.00216 31024454 PMC6465950

[227] Menzies KJ Robinson BH Hood DA. Effect of thyroid hormone on mitochondrial properties and oxidative stress in cells from patients with mtDNA defects. Am J Physiol Cell Physiol. (2009) 296:C355–C62. 10.1152/ajpcell.00415.2007 19036942

[228] Lei J Nowbar S Mariash CN Ingbar DH. Thyroid hormone stimulates Na-K-ATPase activity and its plasma membrane insertion in rat alveolar epithelial cells. Am J Physiol Lung Cell Mol Physiol. (2003) 285:L762–72. 10.1152/ajplung.00376.2002 12740220

[229] Puia G Losi G. Thyroid hormones modulate GABA(A) receptor-mediated currents in hippocampal neurons. Neuropharmacology. (2011) 60:1254–61. 10.1016/j.neuropharm.2010.12.013 21215272

[230] Wiens SC Trudeau VL. Thyroid hormone and gamma-aminobutyric acid (GABA) interactions in neuroendocrine systems. Comp Biochem Physiol A Mol Integr Physiol. (2006) 144:332–44. 10.1016/j.cbpa.2006.01.033 16527506

[231] Davis P Lin H-Y Davis F Luidens M Mousa S Cao J. Molecular basis for certain neuroprotective effects of thyroid hormone. Front Mol Neurosci. (2011) 4:29. 10.3389/fnmol.2011.00029 22016721 PMC3193027

[232] Caria MA Dratman MB Kow LM Mameli O Pavlides C. Thyroid hormone action: nongenomic modulation of neuronal excitability in the hippocampus. J Neuroendocrinol. (2009) 21:98–107. 10.1111/j.1365-2826.2008.01813.x 19076268

[233] Losi G Garzon G Puia G. Nongenomic regulation of glutamatergic neurotransmission in hippocampus by thyroid hormones. Neuroscience. (2008) 151:155–63. 10.1016/j.neuroscience.2007.09.064 18065155

[234] De Vito P Incerpi S Pedersen JZ Luly P Davis FB Davis PJ. Thyroid hormones as modulators of immune activities at the cellular level. Thyroid. (2011) 21:879–90. 10.1089/thy.2010.0429 21745103

[235] Jara EL Munoz-Durango N Llanos C Fardella C Gonzalez PA Bueno SM. Modulating the function of the immune system by thyroid hormones and thyrotropin. Immunol Lett. (2017) 184:76–83. 10.1016/j.imlet.2017.02.010 28216261

[236] van der Spek AH Surovtseva OV Jim KK van Oudenaren A Brouwer MC Vandenbroucke-Grauls C. Regulation of intracellular triiodothyronine is essential for optimal macrophage function. Endocrinology. (2018) 159:2241–52. 10.1210/en.2018-00053 29648626 PMC5920313

[237] Bilal MY Dambaeva S Kwak-Kim J Gilman-Sachs A Beaman KD. A role for iodide and thyroglobulin in modulating the function of human immune cells. Front Immunol. (2017) 8:1573. 10.3389/fimmu.2017.01573 29187856 PMC5694785

[238] Figliozzi RW Chen F Balish M Ajavon A Hsia SV. Thyroid hormone-dependent epigenetic suppression of herpes simplex virus-1 gene expression and viral replication in differentiated neuroendocrine cells. J Neurol Sci. (2014) 346:164–73. 10.1016/j.jns.2014.08.017 25175854 PMC4252976

[239] Vidart J Wajner SM Leite RS Manica A Schaan BD Larsen PR. N-acetylcysteine administration prevents nonthyroidal illness syndrome in patients with acute myocardial infarction: a randomized clinical trial. J Clin Endocrinol Metab. (2014) 99:4537–45. 10.1210/jc.2014-2192 25148231 PMC4255112

[240] Wajner SM Rohenkohl HC Serrano T Maia AL. Sodium selenite supplementation does not fully restore oxidative stress-induced deiodinase dysfunction: implications for the nonthyroidal illness syndrome. Redox Biol. (2015) 6:436–45. 10.1016/j.redox.2015.09.002 26402162 PMC4588414

[241] Berger MM Reymond MJ Shenkin A Rey F Wardle C Cayeux C. Influence of selenium supplements on the post-traumatic alterations of the thyroid axis: a placebo-controlled trial. Intensive Care Med. (2001) 27:91–100. 10.1007/s001340000757 11280679

[242] Corpeno R Dworkin B Cacciani N Salah H Bergman HM Ravara B. Time course analysis of mechanical ventilation-induced diaphragm contractile muscle dysfunction in the rat. J Physiol. (2014) 592:3859–80. 10.1113/jphysiol.2014.277962 25015920 PMC4192708

[243] Corpeno Kalamgi R Salah H Gastaldello S Martinez-Redondo V Ruas JL Fury W. Mechano-signalling pathways in an experimental intensive critical illness myopathy model. J Physiol. (2016) 594:4371–88. 10.1113/JP271973 26990577 PMC4967740

[244] Larsson L Li X Edström L Eriksson LI Zackrisson H Argentini C. Acute quadriplegia and loss of muscle myosin in patients treated with nondepolarizing neuromuscular blocking agents and corticosteroids: mechanisms at the cellular and molecular levels. Crit Care Med. (2000) 28:34–45. 10.1097/00003246-200001000-00006 10667496

[245] Ochala J Gustafson AM Diez ML Renaud G Li M Aare S. Preferential skeletal muscle myosin loss in response to mechanical silencing in a novel rat intensive care unit model: underlying mechanisms. J Physiol. (2011) 589:2007–26. 10.1113/jphysiol.2010.202044 21320889 PMC3090600

[246] Larsson L Friedrich O. Critical Illness Myopathy (CIM) and Ventilator-Induced Diaphragm Muscle Dysfunction (VIDD): acquired myopathies affecting contractile proteins. Compr Physiol. (2016) 7:105–12. 10.1002/cphy.c150054 28135001

[247] Salah H Li M Cacciani N Gastaldello S Ogilvie H Akkad H. The chaperone co-inducer BGP-15 alleviates ventilation-induced diaphragm dysfunction. Sci Transl Med. (2016) 8:350ra103. 10.1126/scitranslmed.aaf7099 27488897

[248] Cacciani N Salah H Li M Akkad H Backeus A Hedstrom Y. Chaperone co-inducer BGP-15 mitigates early contractile dysfunction of the soleus muscle in a rat ICU model. Acta Physiol. (2020) 229:e13425–e. 10.1111/apha.13425 PMC7187345 31799784

[249] Ogilvie H Cacciani N Akkad H Larsson L. Targeting heat shock proteins mitigates ventilator induced diaphragm muscle dysfunction in an age-dependent manner. Front Physiol. (2016) 7:417. 10.3389/fphys.2016.00417 27729867 PMC5037190

[250] Noble EG. Heat shock proteins and their induction with exercise, in Noble EG Locke M editors. Exercise and Stress Response. Boca Raton, FL: CRC Press (2002). p. 49–84. 10.1201/9781420042016

[251] Crul T Toth N Piotto S Literati-Nagy P Tory K Haldimann P. Hydroximic acid derivatives: pleiotropic Hsp co-inducers restoring homeostasis and robustness. Curr Pharm Design. (2013) 19:309–46. 10.2174/138161213804143716 22920902

[252] Addinsall AB Cacciani N Akkad H Salah H Tchkonia T Kirkland JL Larsson L. JAK/STAT inhibition augments soleus muscle function in a rat model of critical illness myopathy via regulation of complement C3/3R. J Physiol. (2021). 10.1113/JP281220. [Epub ahead of print]. 33745126

[253] Salah H Fury W Gromada J Bai Y Tchkonia T Kirkland JL. Muscle-specific differences in expression and phosphorylation of the Janus kinase 2/Signal Transducer and Activator of Transcription 3 following long-term mechanical ventilation and immobilization in rats. Acta Physiol. (2018) 222:e12980. 10.1111/apha.12980 29032602

[254] Bonetto A Aydogdu T Jin X Zhang Z Zhan R Puzis L. JAK/STAT3 pathway inhibition blocks skeletal muscle wasting downstream of IL-6 and in experimental cancer cachexia. Am J Physiol Endocrinol Metab. (2012) 303:E410–21. 10.1152/ajpendo.00039.2012 22669242 PMC3423125

[255] Akkad H Cacciani N Llano-Diez M Corpeno Kalamgi R Tchkonia T Kirkland JL. Vamorolone treatment improves skeletal muscle outcome in a critical illness myopathy rat model. Acta Physiol. (2019) 225:e13172. 10.1111/apha.13172 30120816 PMC8424699

[256] Wood E Hall KH Tate W. Role of mitochondria, oxidative stress and the response to antioxidants in myalgic encephalomyelitis/chronic fatigue syndrome: a possible approach to SARS-CoV-2 'long-haulers'? Chronic Dis Transl Med. (2020) 7:14–26. 10.1016/j.cdtm.2020.11.002 33251031 PMC7680046

[257] Pall ML. Explaining “Unexplained Illnesses”: Disease Paradigm for Chronic Fatigue Syndrome, Multiple Chemical Sensitivity, Fibromyalgia, Post-Traumatic Stress Disorder, Gulf War Syndrome, and Others. Philadelphia, PA: Haworth Press. (2007).

[258] Castro-Marrero J Sáez-Francàs N Segundo MJ Calvo N Faro M Aliste L. Effect of coenzyme Q10 plus nicotinamide adenine dinucleotide supplementation on maximum heart rate after exercise testing in chronic fatigue syndrome-A randomized, controlled, double-blind trial. Clin Nutrition. (2016) 35:826–34. 10.1016/j.clnu.2015.07.010 26212172

[259] Castro-Marrero J Cordero MD Segundo MJ Sáez-Francàs N Calvo N Román-Malo L. Does oral coenzyme Q10 plus NADH supplementation improve fatigue and biochemical parameters in chronic fatigue syndrome? Antioxid Redox Signal. (2015) 22:679–85. 10.1089/ars.2014.6181 25386668 PMC4346380

[260] National Institute of Health. Assessment of N-Acetylcysteine as Therapy for Myalgic Encephalomyelitis/Chronic Fatigue Syndrome (NAC ME/CFS). (2020). Available online at: https://clinicaltrials.gov/ct2/show/NCT04542161 (accessed March 27, 2021).

[261] Kim HG Lee JS Han JM Lee JS Choi MK Son SW. Myelophil attenuates brain oxidative damage by modulating the hypothalamus-pituitary-adrenal (HPA) axis in a chronic cold-stress mouse model. J Ethnopharmacol. (2013) 148:505–14. 10.1016/j.jep.2013.04.046 23665312

[262] Jammes Y Retornaz F. Understanding neuromuscular disorders in chronic fatigue syndrome. F1000Res. (2019) 8:F1000 Faculty Rev-2020. 10.12688/f1000research.18660.1 31814961 PMC6883394

[263] Jammes Y Steinberg JG Delliaux S Brégeon F. Chronic fatigue syndrome combines increased exercise-induced oxidative stress and reduced cytokine and Hsp responses. J Intern Med. (2009) 266:196–206. 10.1111/j.1365-2796.2009.02079.x 19457057

[264] Gerwyn M Maes M. Mechanisms explaining muscle fatigue and muscle pain in patients with Myalgic Encephalomyelitis/Chronic Fatigue Syndrome (ME/CFS): a review of recent findings. Curr Rheumatol Rep. (2017) 19:1. 10.1007/s11926-017-0628-x 28116577

[265] Jammes Y Steinberg JG Delliaux S. Chronic fatigue syndrome: acute infection and history of physical activity affect resting levels and response to exercise of plasma oxidant/antioxidant status and heat shock proteins. J Intern Med. (2012) 272:74–84. 10.1111/j.1365-2796.2011.02488.x 22112145

[266] Klein JR. The immune system as a regulator of thyroid hormone activity. Exp Biol Med. (2006) 231:229–36. 10.1177/153537020623100301 16514168 PMC2768616

[267] van der Poll T Van Zee KJ Endert E Coyle SM Stiles DM Pribble JP. Interleukin-1 receptor blockade does not affect endotoxin-induced changes in plasma thyroid hormone and thyrotropin concentrations in man. J Clin Endocrinol Metab. (1995) 80:1341–6. 10.1210/jcem.80.4.7714108 7714108

[268] Bartalena L Bogazzi F Brogioni S Grasso L Martino E. Role of cytokines in the pathogenesis of the euthyroid sick syndrome. Europ J Endocrinol. (1998) 138:603–14. 10.1530/eje.0.1380603 9678522

[269] Traen S Bochanen N Ieven M Schepens T Bruynseels P Verbrugghe W. Is acyclovir effective among critically ill patients with herpes simplex in the respiratory tract? J Clin Virol. (2014) 60:215–21. 10.1016/j.jcv.2014.04.010 24800905

[270] van den Brink JW Simoons-Smit AM Beishuizen A Girbes AR Strack van Schijndel RJ Groeneveld AB. Respiratory herpes simplex virus type 1 infection/colonisation in the critically ill: marker or mediator? J Clin Virol. (2004) 30:68–72. 10.1016/j.jcv.2003.09.003 15072757

[271] Textoris J Mallet F. Immunosuppression and herpes viral reactivation in intensive care unit patients: one size does not fit all. Crit Care. (2017) 21:230. 10.1186/s13054-017-1803-1 28841888 PMC5574101

[272] Coşkun O Yazici E Sahiner F Karakaş A Kiliç S Tekin M. Cytomegalovirus and Epstein-Barr virus reactivation in the intensive care unit. Med Klin Intensivmed Notfmed. (2017) 112:239–45. 10.1007/s00063-016-0198-0 27435067

[273] Walton AH Muenzer JT Rasche D Boomer JS Sato B Brownstein BH. Reactivation of multiple viruses in patients with sepsis. PLoS ONE. (2014) 9:e98819. 10.1371/journal.pone.0098819 24919177 PMC4053360

[274] Adams Wilson JR Morandi A Girard TD Thompson JL Boomershine CS Shintani AK. The association of the kynurenine pathway of tryptophan metabolism with acute brain dysfunction during critical illness^*^. Crit Care Med. (2012) 40:835–41. 10.1097/CCM.0b013e318236f62d 22080637 PMC3625666

[275] Wang Q Liu D Song P Zou MH. Tryptophan-kynurenine pathway is dysregulated in inflammation, and immune activation. Front Biosci. (2015) 20:1116–43. 10.2741/4363 25961549 PMC4911177

[276] Lögters TT Laryea MD Altrichter J Sokolowski J Cinatl J Reipen J. Increased plasma kynurenine values and kynurenine-tryptophan ratios after major trauma are early indicators for the development of sepsis. Shock. (2009) 32:29–34. 10.1097/SHK.0b013e31819714fa 19060785

[277] Zeden JP Fusch G Holtfreter B Schefold JC Reinke P Domanska G. Excessive tryptophan catabolism along the kynurenine pathway precedes ongoing sepsis in critically ill patients. Anaesth Intensive Care. (2010) 38:307–16. 10.1177/0310057X1003800213 20369765

[278] Dabrowski W Siwicka-Gieroba D Gasinska-Blotniak M Zaid S Jezierska M Pakulski C. Pathomechanisms of non-traumatic acute brain injury in critically ill patients. Medicina. (2020) 56:469. 10.3390/medicina56090469 32933176 PMC7560040

[279] Stone TW Forrest CM Darlington LG. Kynurenine pathway inhibition as a therapeutic strategy for neuroprotection. FEBS J. (2012) 279:1386–97. 10.1111/j.1742-4658.2012.08487.x 22248239

[280] Nguyen DJM Theodoropoulos G Li YY Wu C Sha W Feun LG. Targeting the kynurenine pathway for the treatment of cisplatin-resistant lung cancer. Mol Cancer Res. (2020) 18:105–17. 10.1158/1541-7786.MCR-19-0239 31628200 PMC7262740

[281] Sforzini L Nettis MA Mondelli V Pariante CM. Inflammation in cancer and depression: a starring role for the kynurenine pathway. Psychopharmacology. (2019) 236:2997–3011. 10.1007/s00213-019-05200-8 30806743 PMC6820591

[282] Potter MC Elmer GI Bergeron R Albuquerque EX Guidetti P Wu HQ. Reduction of endogenous kynurenic acid formation enhances extracellular glutamate, hippocampal plasticity, and cognitive behavior. Neuropsychopharmacology. (2010) 35:1734–42. 10.1038/npp.2010.39 20336058 PMC3055476

[283] Englebienne P De Meirleir K. Chronic Fatigue Syndrome: A Biological Approach. Boca Raton, FL: Taylor & Francis (2002). 10.1201/9781420041002

[284] Bolton MJ Chapman BP Van Marwijk H. Low-dose naltrexone as a treatment for chronic fatigue syndrome. BMJ Case Rep. (2020) 13:e232502. 10.1136/bcr-2019-232502 31911410 PMC6954765

[285] Younger J Noor N McCue R Mackey S. Low-dose naltrexone for the treatment of fibromyalgia: findings of a small, randomized, double-blind, placebo-controlled, counterbalanced, crossover trial assessing daily pain levels. Arthritis Rheum. (2013) 65:529–38. 10.1002/art.37734 23359310

[286] Roerink ME Bredie SJ Heijnen M Dinarello CA Knoop H Van der Meer JW. Cytokine inhibition in patients with chronic fatigue syndrome: a randomized trial. Ann Internal Med. (2017) 166:557–64. 10.7326/M16-2391 28265678

[287] Rekeland IG Fosså A Lande A Ktoridou-Valen I Sørland K Holsen M. Intravenous cyclophosphamide in Myalgic Encephalomyelitis/Chronic Fatigue syndrome. an open-label Phase II study. Front Med. (2020) 7:162. 10.3389/fmed.2020.00162 32411717 PMC7201056

[288] Fluge Ø Risa K Lunde S Alme K Rekeland IG Sapkota D. B-Lymphocyte depletion in Myalgic Encephalopathy/ Chronic Fatigue syndrome. an open-label phase II study with rituximab maintenance treatment. PLoS ONE. (2015) 10:e0129898. 10.1371/journal.pone.0129898 26132314 PMC4488509

[289] Tölle M Freitag H Antelmann M Hartwig J Schuchardt M van der Giet M. Myalgic Encephalomyelitis/Chronic Fatigue syndrome: efficacy of repeat immunoadsorption. J Clin Med. (2020) 9:2443. 10.3390/jcm9082443 32751659 PMC7465279

[290] Mitchell WM. Efficacy of rintatolimod in the treatment of chronic fatigue syndrome/myalgic encephalomyelitis (CFS/ME). Expert Rev Clin Pharmacol. (2016) 9:755–70. 10.1586/17512433.2016.1172960 27045557 PMC4917909

[291] Strayer DR Young D Mitchell WM. Effect of disease duration in a randomized Phase III trial of rintatolimod, an immune modulator for Myalgic Encephalomyelitis/Chronic Fatigue Syndrome. PLoS ONE. (2020) 15:e0240403. 10.1371/journal.pone.0240403 33119613 PMC7595369

[292] Diaz-Mitoma F Turgonyi E Kumar A Lim W Larocque L Hyde BM. Clinical improvement in chronic fatigue syndrome is associated with enhanced natural killer cell-mediated cytotoxicity: the results of a pilot study with isoprinosine^®^. J Chronic Fatigue Syndrome. (2003) 11:71–95. 10.1300/J092v11n02_06

[293] Rowe KS. Five-year follow-up of young people with chronic fatigue syndrome following the double blind randomised controlled intravenous gammaglobulin trial. J Chronic Fatigue Syndrome. (1999) 5:97–107. 10.1300/J092v05n03_08

[294] Kogelnik AM Loomis K Hoegh-Petersen M Rosso F Hischier C Montoya JG. Use of valganciclovir in patients with elevated antibody titers against Human Herpesvirus-6 (HHV-6) and Epstein-Barr Virus (EBV) who were experiencing central nervous system dysfunction including long-standing fatigue. J Clin Virol. (2006) 37(Suppl. 1):S33–8. 10.1016/S1386-6532(06)70009-9 17276366

[295] Straus SE Dale JK Tobi M Lawley T Preble O Blaese RM. Acyclovir treatment of the chronic fatigue syndrome. lack of efficacy in a placebo-controlled trial. N Engl J Med. (1988) 319:1692–8. 10.1056/NEJM198812293192602 2849717

[296] Lerner AM Beqaj SH Deeter RG Fitzgerald JT. Valacyclovir treatment in Epstein-Barr virus subset chronic fatigue syndrome: thirty-six months follow-up. In Vivo. (2007) 21:707–13. 18019402

[297] Montoya JG Kogelnik AM Bhangoo M Lunn MR Flamand L Merrihew LE. Randomized clinical trial to evaluate the efficacy and safety of valganciclovir in a subset of patients with chronic fatigue syndrome. J Med Virol. (2013) 85:2101–9. 10.1002/jmv.23713 23959519

[298] Rasa S Nora-Krukle Z Henning N Eliassen E Shikova E Harrer T. Chronic viral infections in myalgic encephalomyelitis/chronic fatigue syndrome (ME/CFS). J Transl Med. (2018) 16:268. 10.1186/s12967-018-1644-y 30285773 PMC6167797

[299] Kerr JR. Epstein-barr virus induced Gene-2 upregulation identifies a particular subtype of Chronic Fatigue Syndrome/Myalgic Encephalomyelitis. Front Pediatr. (2019) 7:59. 10.3389/fped.2019.00059 30918887 PMC6424879

[300] Sepúlveda N Carneiro J Lacerda E Nacul L. Myalgic Encephalomyelitis/Chronic Fatigue syndrome as a hyper-regulated immune system driven by an interplay between regulatory t cells and chronic human herpesvirus infections. Front Immunol. (2019) 10:2684. 10.3389/fimmu.2019.02684 31824487 PMC6883905

[301] Kashi AA Davis RW Phair RD. The IDO metabolic trap hypothesis for the etiology of ME/CFS. Diagnostics. (2019) 9:82. 10.3390/diagnostics9030082 31357483 PMC6787624

[302] Open Medicine Foundation. Kynurenine Trial in ME/CFS. (2020). Available online at: https://www.omf.ngo/2020/08/05/kynurenine-trial-in-me-cfs/ (accessed March 27, 2021).

[303] Schwarcz R Pellicciari R. Manipulation of brain kynurenines: glial targets, neuronal effects, and clinical opportunities. J Pharmacol Exp Ther. (2002) 303:1–10. 10.1124/jpet.102.034439 12235226

[304] Al-Karagholi MA-M Hansen JM Abou-Kassem D Hansted AK Ubhayasekera K Bergquist J. Phase 1 study to access safety, tolerability, pharmacokinetics, and pharmacodynamics of kynurenine in healthy volunteers. Pharmacol Res Perspect. (2020) 9:e00741. 10.1002/prp2.741 33682377 PMC7937944

[305] Jonsjö MA Wicksell RK Holmström L Andreasson A Bileviciute-Ljungar I Olsson GL. Identifying symptom subgroups in patients with ME/CFS - relationships to functioning and quality of life. Fatigue. (2017) 5:33–42. 10.1080/21641846.2017.1287546

[306] Wirth K Scheibenbogen C. A unifying hypothesis of the pathophysiology of Myalgic Encephalomyelitis/Chronic Fatigue Syndrome (ME/CFS): recognitions from the finding of autoantibodies against ß2-adrenergic receptors. Autoimmun Rev. (2020) 19:102527. 10.1016/j.autrev.2020.102527 32247028

[307] Malato J Graça L Nacul L Lacerda E Sepúlveda N. Statistical challenges of investigating a disease with a complex diagnosis. medRxiv. (2021). 10.1101/2021.03.19.21253905

[308] Jason LA Mirin AA. Updating the National Academy of Medicine ME/CFS prevalence and economic impact figures to account for population growth and inflation. Fatigue. (2021) 28:1–5. 10.1080/21641846.2021.1878716

[309] Stanculescu D. “Neither Dying, nor Recovering”: Learning from ICUs to Solve ME/CFS and Fibromyalgia - A Synopsis [Internet]. Health Rising (2019). Available online at: https://www.healthrising.org/blog/2019/11/21/neither-dying-nor-recovering_icus-fibromyalgia-chronic-fatigue-syndrome/ (accessed March 27, 2021).

[310] Stanculescu D. The Relevance of Research on Critical Illnesses for Chronic Fatigue Syndrome ME/CFS: A Vicious Cycle Between Cytokines, Oxidative Stress and Thyroid Hormones [Internet]. Health Rising (2019). Available online at: https://www.healthrising.org/blog/2019/07/24/chronic-fatigue-syndrome-ntis-low-t3-vicious-circle/ (accessed March 27, 2021).

[311] Stanculescu D. Neuroendocrine Dysfunctions in Prolonged Critical Illness: Relevance for Chronic Fatigue Syndrome ME/CFS and Fibromyalgia Pt I [Internet]. Health Rising (2019). Available online at: https://www.healthrising.org/blog/2019/09/05/neuroendocrine-dysfunctions-and-treatments-in-prolonged-critical-illness-relevance-for-chronic-fatigue-syndrome-me-cfs-and-fibromyalgia/ (accessed March 27, 2021).

[312] Stanculescu D. Neuroendocrine Dysfunctions in Prolonged Critical Illness: Relevance for Chronic Fatigue Syndrome ME/CFS and Fibromyalgia Pt II: Treatment [Internet]. Health Rising. (2019). Available online at: https://www.healthrising.org/blog/2019/09/06/neuroendocrine-critical-illness-chronic-fatigue-fibromyalgia-treatment/ (accessed March 27, 2021).

